# Primary Human M2 Macrophage Subtypes Are Distinguishable by Aqueous Metabolite Profiles

**DOI:** 10.3390/ijms25042407

**Published:** 2024-02-18

**Authors:** Amanda L. Fuchs, Stephanann M. Costello, Sage M. Schiller, Brian P. Tripet, Valérie Copié

**Affiliations:** Department of Chemistry and Biochemistry, Montana State University, Bozeman, MT 59717, USA

**Keywords:** M2 macrophage subtypes, M2a, M2b, M2c, M2d, immunometabolism, primary human macrophages, central metabolism, NMR metabolomics, multivariate statistical analysis

## Abstract

The complexity of macrophage (MΦ) plasticity and polarization states, which include classically activated pro-inflammatory (M1) and alternatively activated anti-inflammatory (M2) MΦ phenotypes, is becoming increasingly appreciated. Within the M2 MΦ polarization state, M2a, M2b, M2c, and M2d MΦ subcategories have been defined based on their expression of specific cell surface receptors, secreted cytokines, and specialized immune effector functions. The importance of immunometabolic networks in mediating the function and regulation of MΦ immune responses is also being increasingly recognized, although the exact mechanisms and extent of metabolic modulation of MΦ subtype phenotypes and functions remain incompletely understood. In this study, proton (^1^H) nuclear magnetic resonance (NMR) metabolomics was employed to determine the polar metabolomes of M2 MΦ subtypes and to investigate the relationship between aqueous metabolite profiles and M2 MΦ functional phenotypes. Results from this study demonstrate that M2a MΦs are most distinct from M2b, M2c, and M2d MΦ subtypes, and that M2b MΦs display several metabolic traits associated with an M1-like MΦ phenotype. The significance of metabolome differences for metabolites implicated in glycolysis, the tricarboxylic acid (TCA) cycle, phospholipid metabolism, and creatine–phosphocreatine cycling is discussed. Altogether, this study provides biochemical insights into the role of metabolism in mediating the specialized effector functions of distinct M2 MΦ subtypes and supports the concept of a continuum of macrophage activation states rather than two well-separated and functionally distinct M1/M2 MΦ classes, as originally proposed within a classical M1/M2 MΦ framework.

## 1. Introduction

Human macrophages (MΦs) are fundamental elements of host innate immune responses and provide an immediate line of defense against invasive microbes [1,2,3]. Long recognized to be inherently heterogeneous and versatile, MΦs adapt their functional properties and cellular phenotypes in response to complex microenvironmental cues, including bacterial components, parasites, cytokines, apoptotic cells, immune complexes, and other stimuli [4,5,6,7]. Recent studies have demonstrated that MΦ phenotypes are more complex than the canonical model of M1/M2 polarization, wherein M1 and M2 MΦs were classified as pro- and anti-inflammatory, respectively [8,9]. M1 and M2 MΦs have been reexamined in the context of classical and alternative activation, expanding the subclassification of M2 MΦs into M2a, M2b, M2c, and M2d subtypes, with a remarkable diversity of functions [10,11].

Classically activated M1 MΦs are produced in response to interferon-γ (IFN-γ) stimulation and exposure to bacterial lipopolysaccharide (LPS), and secrete high levels of pro-inflammatory cytokines, including tumor necrosis factor α (TNFα). M1 MΦs produce reactive oxygen species (ROS) through nicotinamide adenine dinucleotide phosphate (NADPH) oxidase activity, contributing to M1 MΦs’ enhanced capabilities to kill intracellular pathogens and exhibit effective anti-tumor activities [5,8,12].

In contrast, M2 MΦs are highly phenotypically and functionally heterogeneous, playing prominent roles in diverse anti-inflammatory responses, acting in pathogen defense, apoptotic cell clearance, wound healing, organ morphogenesis, tissue turnover, and endocrine signaling [10]. M2 MΦ subtypes, including M2a, M2b, M2c, and M2d, have been defined based on gene transcription profiles and cell surface marker expression [10].

M2a MΦs, also known as wound-healing MΦs, are produced following stimulation by interleukin-4 (IL-4) and interleukin-13 (IL-13). They are characterized by high expression of mannose receptor (MR or CD206) protein and decoy interleukin-1 receptor (IL-1R), and secrete pro-fibrotic factors such as transforming growth factor beta (TGF-β), insulin growth factor (IGF), and fibronectin, which are all important components of wound healing and tissue repair [10,13]. 

M2b MΦs, also called regulatory MΦs, are induced by exposure to immune complexes (ICs), bacterial LPS, toll-like receptors (TLRs), or IL-1R ligands [7,10]. M2b MΦs regulate a wide range of immune and inflammatory responses, and in cancer and infectious diseases can promote tumors and the spread of infections by reducing immune and inflammatory responses [14]. 

Although M2 MΦs are generally considered to be anti-inflammatory due to their ability to produce high levels of interleukin-10 (IL-10) and low levels of interleukin-12 (IL-12), M2b MΦs are an exception, as they produce high levels of pro-inflammatory cytokines, such as interleukin-1β (IL-1β), interleukin-6 (IL-6), and TNFα, in addition to producing high levels of IL-10 and low levels of IL-12 [14,15].

M2c MΦs, referred to as deactivated M2c MΦs in reference to these MΦs’ ability to adopt M2 activation phenotypes following M1 activation in vitro, i.e., reprogramming of M1-like MΦs into M2 MΦs, are induced by IL-10 and glucocorticoids, and, similar to M2b MΦs, are classified as regulatory macrophages [7,13]. M2c MΦs exhibit strong anti-inflammatory activities and are involved in the effective clearance of apoptotic cells, expressing high levels of the Mer receptor tyrosine kinase (MerTK) and releasing significant levels of the anti-inflammatory cytokines IL-10 and TGF-β [5,13]. 

Lastly, M2d MΦs are produced following co-stimulation with TLR and adenosine A2A receptor agonists and are characterized by expression and secretion of high levels of IL-10 and vascular endothelial growth factor (VEGF), and low levels of TNFα and IL-12 [13]. These characteristics impart M2d MΦs with pro-angiogenic properties, resembling features of tumor associated MΦs (TAMs) [5]. M2d MΦs differ, however, from other M2 MΦ subtypes by not expressing high levels of mannose receptor (MR) [13].

Despite the identification of characteristic features of M2 MΦ subtypes, there remains significant questions regarding the nature, metabolism, and functional characteristics of these different M2 MΦ subtypes. It is now increasingly recognized that MΦ activation states and cellular phenotypes are closely linked to their metabolic characteristics [16]. Numerous studies have demonstrated the importance of immunometabolic regulatory networks in mediating immune cell responses to environmental cues, and metabolic changes are viewed as critical elements of MΦ effector function [2,17,18,19]. 

The current study investigated the metabolic traits of M2a, M2b, M2c, and M2d MΦ subtypes generated from primary human monocytes, using proton (^1^H) nuclear magnetic resonance (NMR) metabolomics methods. Results from this study reveal that M2 MΦ subtypes exhibit very different characteristic polar, i.e., water-soluble, metabolite profiles that correlate closely with their M2 MΦ cellular phenotypes and activated functional states.

## 2. Results

### 2.1. Quantitative Metabolic Profiles Reveal M2a MΦs Are Remarkably Distinct from Other M2 MΦ Subtypes

**Figure 1:**
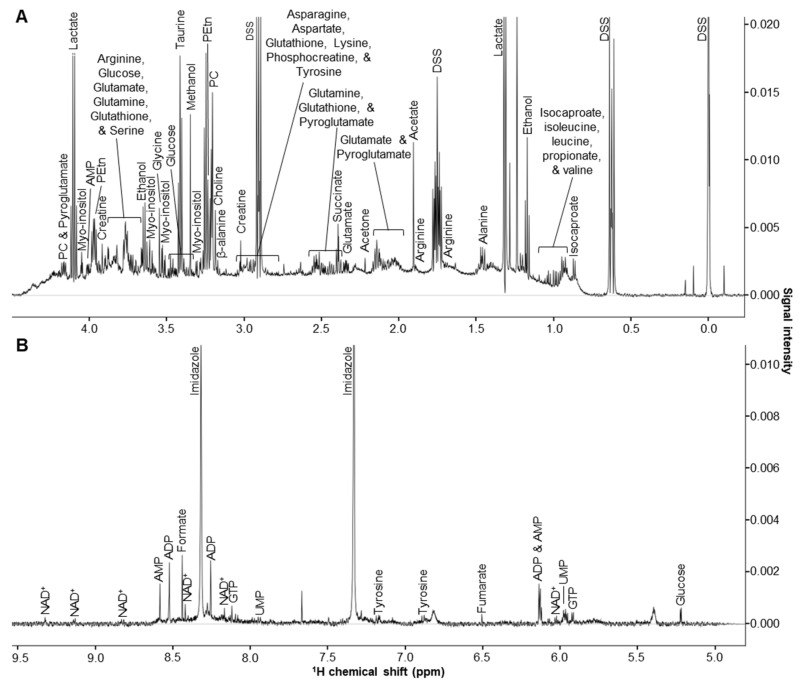
Representative 1D ^1^H NMR spectrum of intracellular metabolite extracts obtained from M2 MΦ subtypes. ^1^H chemical shift regions corresponding to (**A**) 0.0 to ~4.5 ppm and (**B**) 5.0 to ~9.5 ppm are depicted. Abbreviations denote: ADP, adenosine diphosphate; AMP, adenosine monophosphate; DSS, 4,4-dimethyl-4-silapentane-1-sulfonic acid; GTP, guanosine triphosphate; NAD^+^, nicotinamide adenine dinucleotide; and UMP, uridine monophosphate.

Untargeted 1D ^1^H NMR metabolomics methods were used to characterize the polar metabolite profiles of primary human M2 MΦ subtypes, including M2a, M2b, M2c, and M2d MΦs. Representative ^1^H NMR spectra from intra- and extracellular metabolite extracts obtained from M2 MΦ subtype cell samples are shown in Figure 1 and Figure S2, respectively. Examination of characteristic ^1^H chemical shifts, spectral patterns, and NMR signal intensities led to the identification and quantitation of 54 intra- and 51 extracellular metabolites for each M2 MΦ subtype (Tables S1 and S2).

**Figure 2:**
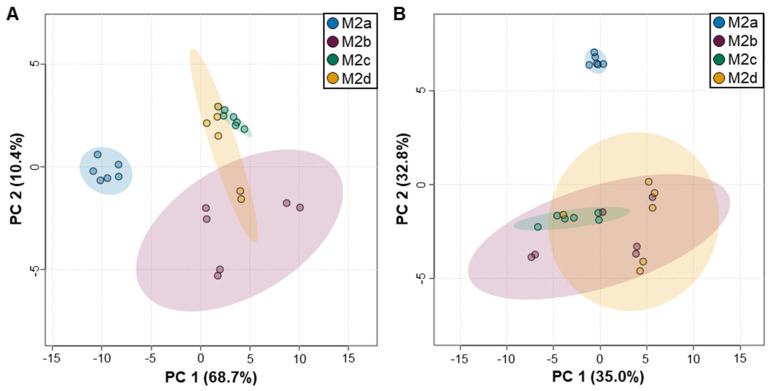
2D-PCA scores plots of M2a, M2b, M2c, and M2d MΦ subtype (**A**) intra- and (**B**) extracellular polar metabolite profiles. Shaded ellipses indicate corresponding 95% confidence intervals. Blue colored circles denote M2a MΦs; red denotes M2b MΦs; green denotes M2c MΦs; and orange denotes M2d MΦs.

2D-PCA analysis was employed to assess whether M2 MΦ subtypes were distinguishable from one another based upon distinct, characteristic intra- and extracellular polar metabolite profiles. This analysis revealed that M2a MΦs were strikingly different from the other M2 MΦ subtypes and separated primarily along PC1 of the 2D-PCA scores plots obtained for both intra- (Figure 2A) and extracellular (Figure 2B) datasets, with PC1 and PC2 accounting for 79.1% and 67.8% of the sample variance for intra- and extracellular metabolite profiles, respectively. 

2D PLS-DA scores plots enabled the visualization of specific metabolites whose pattern differences contributed to the distinct clustering of the M2 MΦ subtypes based on characteristic intra- and extracellular metabolite profiles (Figure S3). Validation tests including R2, Q2, separation distance permutation test, CER, and AUROC analyses were performed to verify that the group separation observed in the supervised PLS-DA models for both intra- and extracellular datasets was real. Results from these PLS-DA model validation tests are included as Supplementary Information (Figures S4 and S5) [20]. These analyses demonstrated that both PLS-DA models for intra- and extracellular metabolite profiles are valid and not over fit, and clearly demonstrated that M2a MΦs are metabolically distinct from the other M2 MΦ subtypes.

VIP scores plots associated with the PLS-DA analyses (Figure S6) were evaluated to further interrogate which metabolites contribute most to the separation of the M2 MΦ subtypes for both the intra- and extracellular polar metabolite profiles. VIP scores ≥ 1.2 were considered valuable discriminators and significant contributors to the separation observed along component 1 or 2. For the intracellular metabolite dataset associated with the different M2 MΦ subtypes, 15 unique metabolites exhibited VIP scores ≥ 1.2 across both components 1 and 2, and included glycerophosphocholine, phosphatidyl ethanolamine, choline, taurine, AMP (adenosine monophosphate), GTP (guanosine triphosphate), G1P (glucose-1-phosphate), phosphocreatine, histidine, urea, phenylalanine, lysine, hypoxanthine, β-alanine, and glutamate. Of these, all were decreased in concentration in the intracellular metabolite profiles of M2a MΦs relative to the levels measured in the M2b, M2c, and M2d subtypes, except for AMP and phosphocreatine, which exhibited levels comparable to those measured for the M2b MΦ subtype. Levels of AMP were decreased, and phosphocreatine elevated in the M2a and M2b MΦ subtypes relative to both the M2c and M2d MΦ subtypes (Figure S6).

In the analysis of the extracellular polar metabolite profiles, VIP scores plots associated with both components 1 and 2 yielded 15 metabolites with VIP scores ≥ 1.2, and included pyruvate, betaine, allantoin, 2-HIB (2-hydroxyisobutyrate), ketoisoleucine, formate, threonine, glutamine, phosphocholine, propionate, fructose, 3-HIB (3-hydroxyisobutyrate), malate, ketoleucine, tryptophan, lactate, and 3-HB (3-hydroxybutyrate). Extracellular metabolite patterns were more variable than the intracellular metabolite profiles determined for all M2 MΦ subtypes, with the M2a and M2b MΦ subtypes often trending with similar relative concentrations compared with those measured for the M2c and M2d MΦ subtypes. 

**Figure 3:**
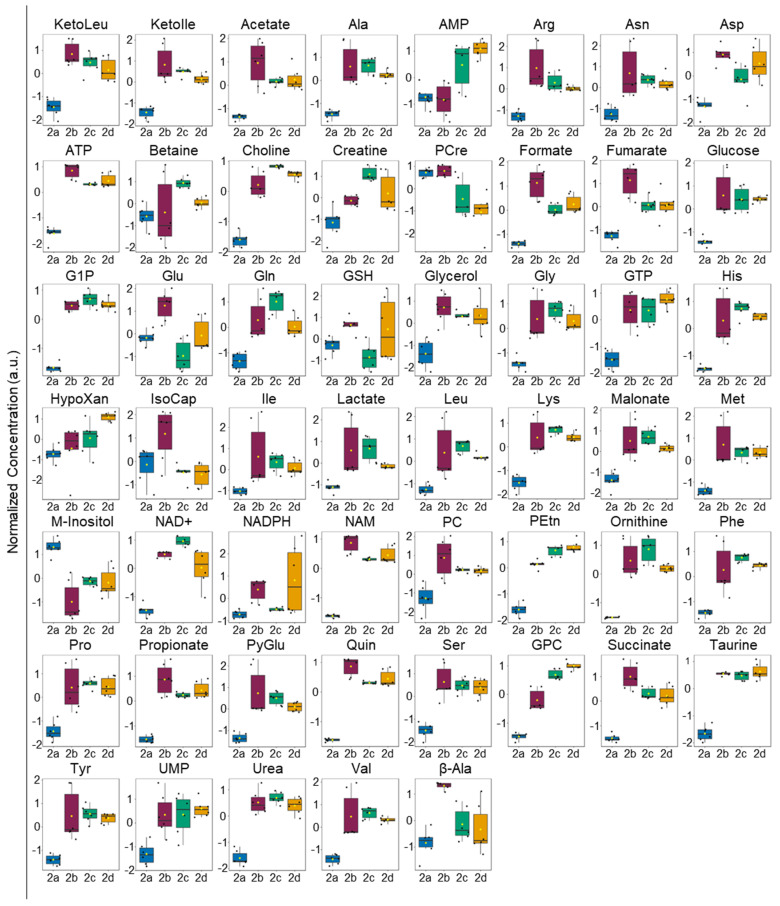
One-way parametric ANOVA analysis of intracellular metabolite levels identified 53 significant metabolites (*p* < 0.05). Analysis performed using Tukey’s post-hoc analysis. The box and whisker plots display the data distribution, with each black dot representing a sample, and black lines indicating the median. First and third quartiles are represented in each filled box diagram, and whiskers extending from each quartile represent the maximum or minimum value. Abbreviations: KetoLeu, ketoleucine; KetoIle, ketoisoleucine; Ala, alanine; AMP, adenosine monophosphate; Arg, arginine; Asn, asparagine; ATP, adenosine triphosphate; PCre, phosphocreatine; G1P, glucose-1-phosphate; Glu, glutamate; Gln, glutamine; GSH, glutathione; Gly, glycine; GTP, guanosine triphosphate; His, histidine; HypoXan, hypoxanthine; IsoCap, isocaproate; Ile, isoleucine; Leu, leucine; Lys, lysine; Met, methionine; M-Inositol, myo-inositol; NAD, nicotinamide adenine dinucleotide; NADPH, nicotinamide adenine dinucleotide phosphate; NAM, niacinamide; PC, phosphocholine; PEtn, phosphatidyl ethanolamine; Phe, phenylalanine; Pro, proline; PyGlu, pyroglutamate; Quin, quinolinate; Ser, serine; GPC, glycerophosphocholine; Tyr, tyrosine; Val, valine; and β-Ala, β-alanine.

**Figure 4:**
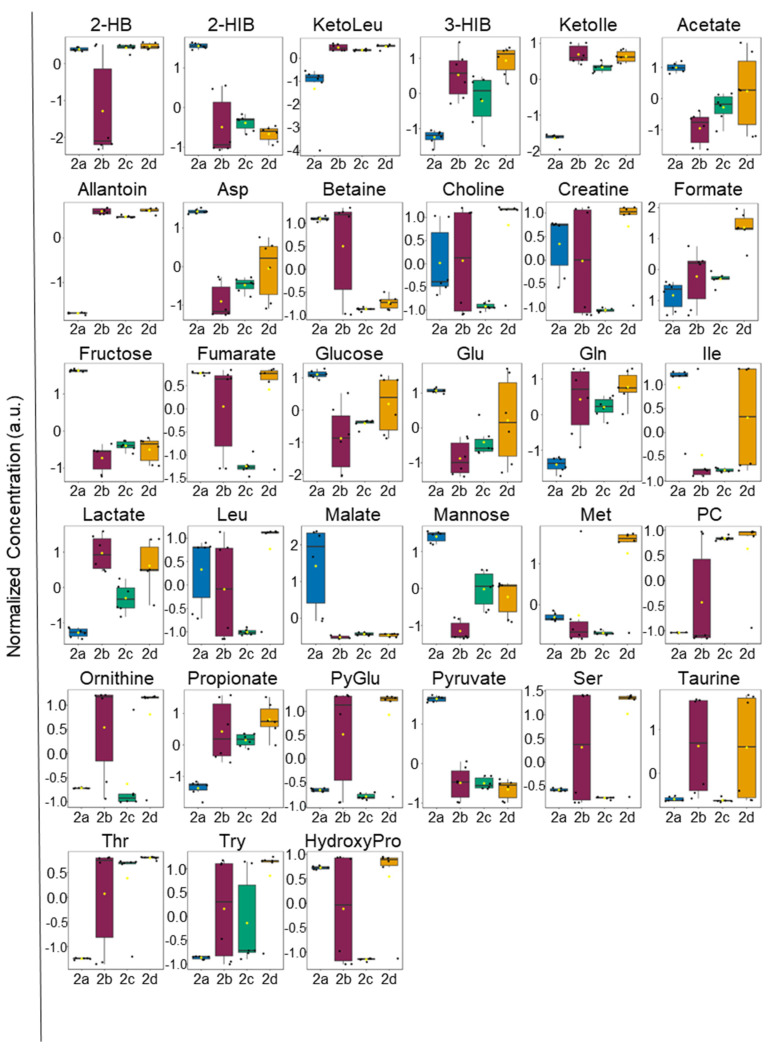
One-way parametric ANOVA analysis of extracellular metabolite levels identified 33 significant metabolites (*p* < 0.05). Analysis performed using Tukey’s honestly significant difference (HSD) post-hoc analysis. The box and whisker plots display the data distribution, with each black dot representing a sample, and black lines indicating the median. First and third quartiles are represented in each filled box diagram, and whiskers extending from each quartile represent the maximum or minimum value. Abbreviations: 2-HB, 2-hydroxybutyrate; 2-HIB, 2-hydroxyisobutyrate; KetoLeu, ketoleucine; 3-HIB, 3-hydroxyisobutyrate; KetoIle, ketoisoleucine; Asp, aspartate; Glu, glutamate; Gln, glutamine; Ile, isoleucine; Leu, leucine; Met, methionine; PC, phosphocholine; PyGlu, pyroglutamate; Ser, serine; Thr, threonine; Tyr, tyrosine; and HydroxyPro, hydroxyproline.

Metabolic differences between the M2 MΦ subtypes were also readily apparent in HCA and heatmap visualizations of the intra- and extracellular metabolite profiles (Figures S7A and S7B, respectively) and ANOVA plot (Figure 3 and Table S3, and Figure 4 and Table S4, respectively). As observed with the VIP scores plots, intracellular polar metabolite concentrations were significantly reduced in M2a MΦs relative to other M2 MΦ subtypes across nearly all identified metabolites, apart from myo-inositol and phosphocreatine (Figure 3 and Figure S7A, and Table S3). Differences in extracellular polar metabolite profiles could not distinguish M2b MΦs from either M2c or M2d MΦs when hierarchically clustered (Figure S7B); however, M2a MΦs were again clearly disparate from all other M2 MΦ subtypes. Several extracellular metabolites were lower in concentration in M2a MΦ cultures relative to other M2 MΦ subtypes, including 3-HIB, allantoin, formate, lactate, glutamine, ketoisoleucine, ketoleucine, methionine, phosphocholine, propionate, pyroglutamate, and threonine. In contrast, a subset of extracellular metabolites displayed higher concentrations in M2a MΦ cultures relative to other M2 MΦ subtypes, and included 2-hydroxybutyrate (2-HB), 2-HIB, acetate, aspartate, betaine, fructose, fumarate, glucose, glutamate, hydroxyproline, malate, mannose, and pyruvate (Figure 4 and Figure S7B, and Table S4).

Since M2a MΦs exhibited polar metabolomes that were strikingly distinct from the other M2 MΦ subtypes, which could have masked important metabolic pattern differences between M2b, M2c, and M2d MΦs, additional multivariate statistical analyses were focused only on investigating metabolic pattern differences between M2b, M2c, and M2d MΦs, without inclusion of M2a MΦ metabolomics information.

### 2.2. Unique Intracellular Metabolite Signatures Reveal Significant Metabolic Traits Associated with M2b, M2c, and M2d MΦ Subtypes

**Figure 5:**
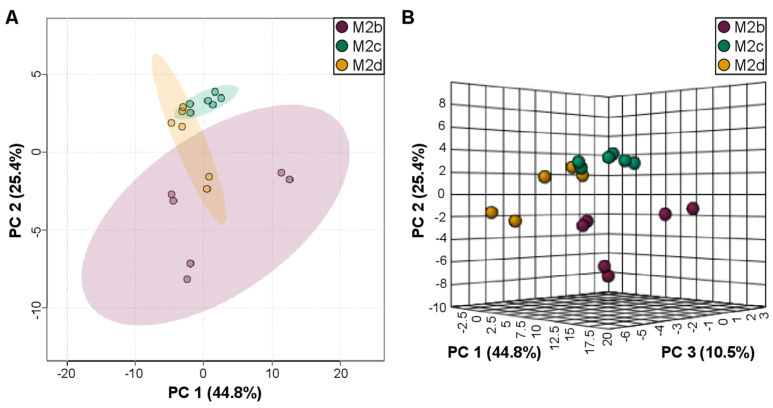
2D (**A**) and 3D PCA (**B**) scores plots highlight the separate classification of the M2b, M2c, and M2d MΦ subtypes, resulting from their distinct intracellular polar metabolite profiles. (**A**) 2D-PCA scores plot highlighting the separate clustering of the M2b MΦ group (purple) from the M2c (green) and M2d (orange) MΦ subtypes, with PC1 and PC2 accounting for 70.2% of the variance, and shaded ellipses representing the 95% confidence intervals of each group. (**B**) 3D PCA scores plot clearly separating the three different M2b (purple), M2c (green), and M2d (orange) MΦ subtypes from each other, with PC1, PC2, and PC3 accounting for a total of 80.7% of the variance.

Separation between M2b, M2c, and M2d MΦ subtypes was observed (Figure 5) when modeling their intracellular metabolite profiles using unsupervised PCA analysis, revealing separate clustering of M2b, M2c, and M2d MΦ subtypes (Figure 5). The M2b MΦ group clustered distinctly from the M2c and M2d MΦ subtypes, most notably along PC2 of the PCA scores plot (Figure 5A,B). While M2b MΦs were the most distinct from the M2c and M2d MΦ subgroups, they also exhibited greater intra-group metabolite profile variability, as observed by the spread of the data for different samples within the M2b MΦ group in both the 2D and 3D PCA scores plots (Figure 5A and Figure 5B, respectively).

The M2c and M2d MΦ subgroups clustered closer in the PCA ordination space, suggesting greater intracellular metabolite profile similarity between these two groups, compared with the M2b MΦ subtype. PC2 accounted for 25.4% of variance and resulted in the largest separation of the three M2b, M2c, and M2d MΦ subtypes along this principal component of the 2D-PCA scores plot. It should also be taken into consideration that PC3, which accounted for an additional 10.5% of the variance, clearly separated and distinguished the M2b, M2c, and M2d MΦ subtypes from each other, as evidenced by their unique water-soluble metabolite patterns.

**Figure 6:**
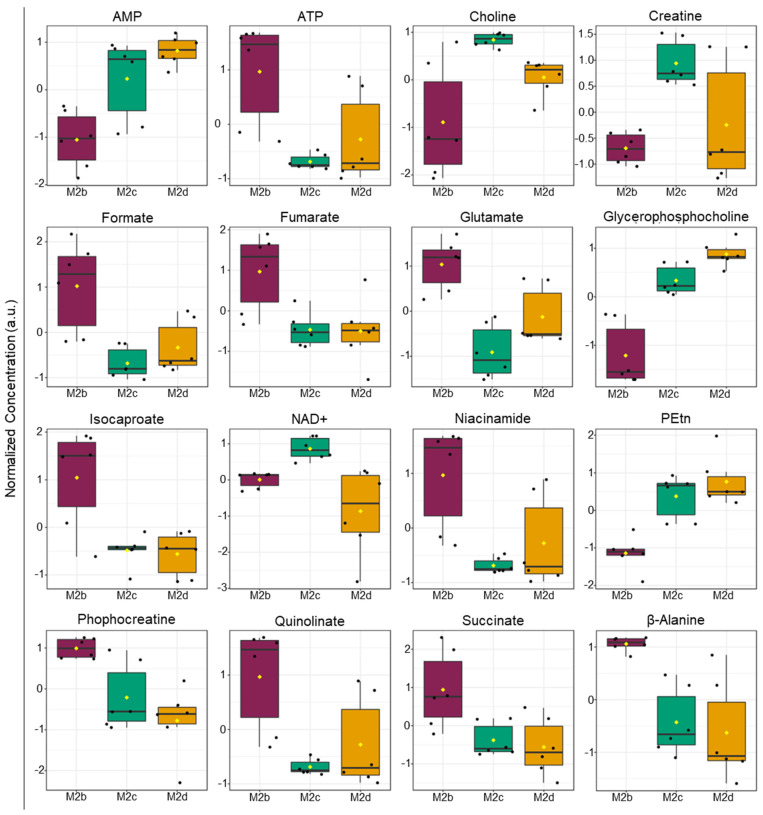
One-way parametric ANOVA analysis of intracellular metabolite levels of M2b, M2c, and M2d MΦs identified 16 significant metabolites (*p* < 0.05). Analysis performed using Tukey’s honestly significant difference (HSD) post-hoc analysis. The box and whisker plots display the data distribution, with each black dot representing a sample, and black lines indicating the median. First and third quartiles are represented in each filled box diagram, and whiskers extending from each quartile represent the maximum or minimum value. Abbreviations: AMP, adenosine monophosphate; ATP, adenosine triphosphate; NAD+, nicotinamide adenine dinucleotide; and PEtn, phosphatidyl ethanolamine.

An ANOVA analysis was performed to assess the extent of group-wise variation for the intracellular metabolite patterns determined for the M2b, M2c, and M2d MΦ subtypes. This analysis identified 16 intracellular metabolites that were significant (*p* < 0.05) discriminators of the three different M2 MΦ subtypes (Figure 6 and Table S5), and included AMP, ATP, choline, creatine, formate, fumarate, glutamate, GPC, isocaproate, NAD+, niacinamide, PEtn, phosphocreatine, quinolinate, succinate, and β-alanine.

From this analysis, the intracellular metabolite profiles of the M2b MΦs differed significantly from those of the M2c and M2d MΦs. In contrast, the intracellular metabolite patterns of the M2c and M2d MΦs were more comparable to each other. Metabolites with elevated levels measured in M2b compared with M2c and M2d MΦs included adenosine triphosphate (ATP), phosphocreatine, formate, fumarate, glutamate, isocaproate, niacinamide, quinolinate, succinate, and β-alanine. Metabolites that were lower in concentration in M2b MΦs compared with the M2c and M2d MΦ subtypes included AMP, choline, creatine, nicotinamide adenine dinucleotide (NAD^+^), phosphatidyl ethanolamine, and glycerophosphocholine. 

In summary, these analyses revealed that the intracellular metabolic profiles of the M2b MΦ subtype differ most significantly from those measured for the M2c MΦ subtype, as shown by the larger ordination space observed in the PCA (Figure 5) and the ANOVA analysis (Figure 6 and Table S5). The intracellular metabolomes of M2b and M2d MΦ subtypes clustered more closely in the PCA scores plots, with fewer significant metabolites discriminating between the two groups. Lastly, the M2c and M2d MΦ subtypes appeared to group more closely together, because the two MΦ clusters overlap to a greater extent in the 3D PCA scores plot (Figure 5B), and only one metabolite (NAD+) was found to be a significant discriminator between these two M2 MΦ subtypes in the ANOVA analysis (Figure 6 and Table S5).

### 2.3. Variability in Extracellular Metabolite Profiles of M2b, M2c, and M2d MΦ Subtypes Does Not Occlude the Ability to Separate These M2 MΦ Subtypes Based upon Characteristic Extracellular Water Soluble Metabolite Patterns

**Figure 7:**
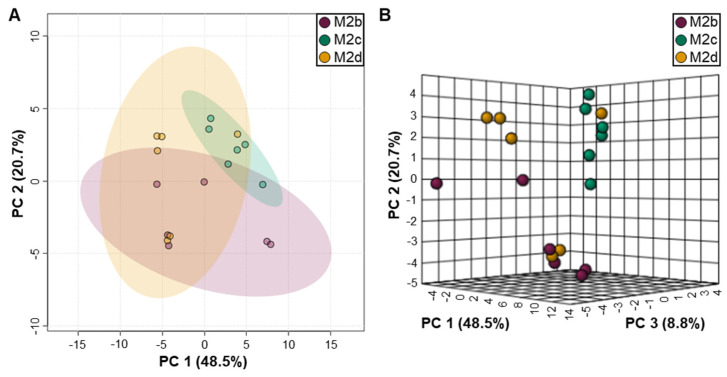
2D (**A**) and 3D (**B**) PCA scores plots generated from the extracellular polar metabolite profiles of M2 MΦ subtypes, including M2b (purple), M2c (green), and M2d (orange). In (**A**), shaded ellipses depict the 95% confidence intervals for the classification of the different M2 MΦ subtypes into distinct groups, based upon characteristic differences in extracellular metabolite profiles, with PC1 and PC2 accounting for 69.2% of the variance. Whereas in (**B**), the three principal components delineating the axes of the 3D PCA scores plot account for 78% of the variance of the PCA model, which clearly depicts the clearer separation of M2c MΦs from M2b and M2d MΦs.

Analysis of polar extracellular metabolites quantified from the spent cell culture medium of the different M2 MΦ cell cultures led to the identification of 51 extracellular metabolites (Table S2). 2D- and 3D PCA scores plots generated from statistically normalized extracellular metabolite concentrations demonstrated that the extracellular metabolite profiles of the M2 MΦ subtypes are more variable compared with the measured intracellular metabolite levels (Figure 7). Variations in the profiling and quantitation of extracellular metabolites within groups yielded less evident distinctions between M2 MΦ subtypes and more overlap between the M2 MΦ subgroups, at least with regards to unsupervised PCA analysis, and 2D- and 3D PCA scores plots (Figure 7).

Unlike the intracellular metabolite datasets, the extracellular metabolite profiles of the M2b and M2d MΦ subtypes yielded greater overlap between these two groups in the PCA ordination space (Figure 7A,B), suggesting greater similarity between these two M2 MΦ subtypes. The extracellular metabolite patterns of the M2c MΦs were the most distinct, as individual samples of the M2c MΦ subgroup clustered closer together and overlapped less with the extracellular metabolite profiles measured for the M2b and M2d MΦ samples. These grouping patterns were most apparent when examining the 3D PCA scores plot (Figure 7B), whereby PC1, PC2, and PC3 accounted for a total of 78% of the sample variance.

**Figure 8:**
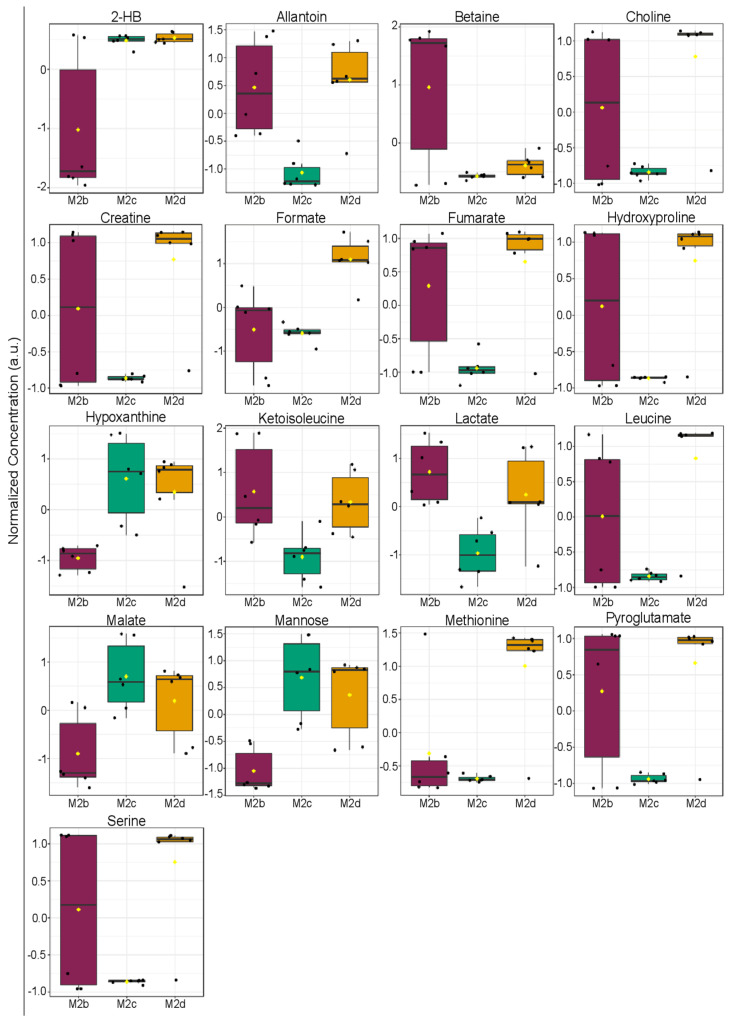
One-way parametric ANOVA analysis of extracellular metabolite levels quantified in M2b (purple), M2c (green), and M2d (orange) MΦ subtypes. This analysis identified 17 extracellular metabolites that were significant (*p* < 0.05) discriminators of the three M2 MΦ subtypes. The ANOVA was performed using Tukey’s HSD post-hoc analysis. Black dots represent samples, and the box and whisker plots display the median (black line in the box), first and third quartiles, range, and mean (denoted by a yellow diamond symbol in each box).

ANOVA analyses of the extracellular polar metabolite patterns measured in M2b, M2c, and M2d MΦs identified 17 significant metabolites (*p* < 0.05) from a total of 51 extracellular metabolites that were identified and quantified in spent cell culture media (Figure 8 and Table S6). These 17 extracellular metabolites included 2-HB, allantoin, betaine, choline, creatine, formate, fumarate, hydroxyproline, hypoxanthine, ketoisoleucine, lactate, leucine, malate, mannose, methionine, pyroglutamate, and serine. Notably, very few amino acids were included in this list of significant extracellular metabolites. The largest variability in extracellular metabolite concentrations was observed for the M2b MΦ group. Yet, trends could still be identified in which levels of 2-HB, malate, mannose, and methionine were lower in M2b MΦs compared with the levels measured in the spent media of M2c and M2d MΦs.

Interesting patterns were also observed in the ANOVA plot shown in Figure 8 and Table S6 when examining differences in extracellular metabolite levels between M2c and M2d MΦs. Several metabolites were lower in concentration in the M2c MΦs compared with M2d MΦs, and included ketoisoleucine, allantoin, choline, creatine, formate, fumarate, lactate, leucine, methionine, pyroglutamate, serine, and hydroxyproline. The remaining four metabolites identified as significant in the ANOVA analysis, 2-HB, hypoxanthine, malate, and mannose, exhibited comparable levels in both the M2c and M2d MΦ subgroups (Figure 8 and Table S6).

### 2.4. Analysis of M2b, M2c, and M2d Intra- and Extracellular Metabolomics Datasets Identified Shared Metabolites as Significant Discriminators of M2 MΦ Subtypes

**Figure 9:**
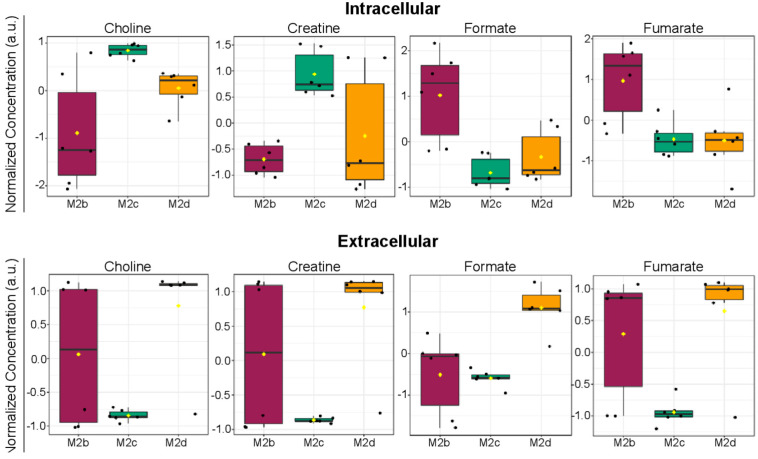
Four metabolites identified in both the intra- and extracellular datasets of M2b, M2c, and M2d MΦs found to be significant (*p* < 0.05) discriminators between M2b, M2c, and M2d MΦ subtypes.

When comparing intra- and extracellular metabolites that were potentially significant discriminators of M2b, M2c, and M2d MΦ subtypes, four were found to be common to both intra- and extracellular datasets, and included choline, creatine, formate, and fumarate (Figure 9).

Interestingly, intracellular choline levels were elevated in M2c MΦs relative to M2b MΦs, whereas extracellular choline levels were lower in the M2c MΦs compared with the extracellular levels measured in the M2d MΦ subtype.

Intracellular creatine levels were significantly lower in M2b MΦs relative to M2c MΦs, whereas intracellular formate and fumarate levels were significantly higher in M2b MΦs relative to M2c MΦs. Intracellular levels of creatine, formate, and fumarate were comparable in both M2c and M2d MΦs. 

When examining the extracellular metabolite profiles of the M2b, M2c, and M2d MΦ subtypes, significant variability was measured in the extracellular profiles of the M2b MΦs that occluded a detailed analysis of similarities and differences between the M2b MΦ group and either the M2c or M2d MΦ subtypes. However, examination of the extracellular profiles of the M2c and M2d MΦ subtypes revealed compelling differences in the extracellular levels of choline, creatine, formate, and fumarate, all found to be elevated in M2d MΦs (Figure 9).

## 3. Discussion

M2 MΦs, often referred to as anti-inflammatory or alternatively activated MΦs, are recognized for their abilities to attenuate M1 pro-inflammatory responses, clear apoptotic cells, and promote and regulate wound-healing processes [19,21,22,23]. Essential metabolic pathways closely associated with the M2 MΦ polarization state include preferential use of the tricarboxylic acid (TCA) cycle coupled with oxidative phosphorylation (OXPHOS) for energy production, fatty acid oxidation (FAO) that can fuel OXPHOS, and the arginase (ARG1) reaction that produces ornithine for downstream wound-resolving biosynthetic reactions [21,24,25,26,27,28]. Within the entire class of M2 MΦs exist the subtypes M2a, M2b, M2c, and M2d, each exhibiting a uniquely specialized function to coordinate various biological processes associated with activated M2 MΦ states. M2a MΦs have been reported to facilitate wound healing, while M2b MΦs regulate the extent of an inflammatory response. M2c MΦs phagocytose apoptotic cells, and M2d MΦs promote angiogenesis and tumor progression [19,21,29,30]. The molecular characterization of the immunometabolic networks underlying the function of the different M2 MΦ subclasses is still a nascent field of study [23,29]. Considering our limited knowledge, and the uniquely specialized function of each M2 MΦ subtype, results from this study enhance our understanding of the metabolic distinctions that are unique to each M2 MΦ subtype and that potentially mediate M2 MΦ biological effector functions. 

Proteomic analyses conducted by Pengfei et al. used the human-derived leukemia monocytic cell line known as THP-1 (Tohoku Hospital Pediatrics-1) to derive and evaluate M2a, M2b, M2c, and M2d MΦs, and determined that M2a MΦs are more similar to M2c MΦs and that M2b MΦs are more similar to M2d MΦs, based on each MΦ subtype’s protein expression profiles [29]. In contrast, a mass spectrometry (MS)-based metabolomics and transcriptomic analysis study suggested that M2a MΦs are functionally distinct from M2b, M2c, and M2d MΦs, as inferred from distinct nonpolar metabolite signatures associated with physiological characteristics, which include M2a MΦs’ enhanced phagocytic and proliferative capabilities, together with their reduced role in promoting angiogenesis and extracellular matrix remodeling when compared with other M2 MΦ subtypes [23]. This analysis also suggested that omics patterns of M2a, M2c, and M2d MΦ subtypes are consistent with tissue repair functions, and that M2b MΦs are most distinct from all other M2 MΦ subtypes and closer to an M1 MΦ phenotype [23]. 

The present metabolomics study identified characteristic polar metabolite patterns, i.e., metabotypes, associated with distinct primary human M2a, M2b, M2c, and M2d MΦs, despite M2 MΦ samples being generated from multiple blood donor volunteers. Analysis of both intra- and extracellular polar metabolites indicated that the M2a MΦ subtype metabolome is vastly different from the metabolomes of M2b, M2c, and M2d MΦ subtypes. Significant metabolite differences between M2b, M2c, and M2d MΦs were also observed, and resulted in the distinct and separate clustering of each M2 MΦ subtype following multivariate statistical analysis of their polar metabolite profiles. The significance of these findings is further discussed below.

### 3.1. Distinct Elevation of Myo-Inositol in M2a MΦs Is Chiefly Attributable to Its Specialized Function

Examination of the polar metabolite profiles of the different M2 MΦ subtypes indicated that most intracellular metabolite levels are significantly lower (FDR *p* ≤ 0.05) in M2a MΦs compared with levels measured in the other M2 MΦ subtypes (Figure 3 and Table S3), except for myo-inositol, whose intracellular levels are highest in M2a MΦs.

Myo-inositol is implicated in improving MΦ respiratory burst and wound healing [31], the latter being an established function of M2a MΦs. Phagocytosis, a mechanism more closely associated with M2c macrophages, is also promoted by myo-inositol, as this metabolite induces Rac Family Small GTPase 2 (RAC2) mRNA and protein expression, which prompts ROS production and depolarization of the MΦ membrane potential [31]. Though not significantly different among the M2b, M2c, and M2d MΦ subtypes, M2c MΦs exhibited the second highest average intracellular level of myo-inositol, which suggests that myo-inositol levels are characteristic of a phagocytic phenotype.

As mentioned, M2a MΦs are known to facilitate wound healing through enhancing cell growth and tissue repair, whereas M2b, M2c, and M2d MΦs act in regulating inflammation, phagocytosis, and tumorigenesis, respectively [14,21]. Many metabolite signatures identified in our ANOVA analyses differentiate M2a MΦs from the other M2 MΦs based upon lower energetics, i.e., increased ATP needs resulting from M2a MΦ immunological effector functions. These data demonstrate that different M2 MΦ subtypes rely preferentially either on anaerobic glycolysis, or aerobic TCA cycle coupled with oxidative phosphorylation (OXPHOS) activity for energy (ATP) production.

### 3.2. Metabolites Involved in Glycolysis, the TCA Cycle, and OXPHOS Differentiate M2a MΦs from M2b, M2c, and M2d MΦ Subtypes, Suggesting That Metabolome Differences Report on M2 vs. M1-like MΦ Phenotypic Distinctions

Increased glycolysis and lactate production are characteristic markers of MΦ activation, regardless of polarization state, although these markers are most indicative of an M1 polarization state, as this catabolic pathway is attributed to enabling phagocytosis, pro-inflammatory cytokine secretion, and ROS production [19]. Based on the significantly (FDR *p* ≤ 0.05) altered metabolite levels identified in this study, M2b, M2c, and M2d MΦ subtypes could be classified as exhibiting more of an M1-like, or pro-inflammatory phenotype compared with M2a MΦs, given observed metabolite level changes suggesting enhanced glycolysis in M2b, M2c, and M2d MΦs compared with M2a MΦs. 

The metabolomics signatures of the M2a MΦs observed in this study more closely mirror the classical M2 MΦ anti-inflammatory polarization state associated with the aerobic TCA cycle and OXPHOS activity. Indeed, this study found that M2a MΦs have significantly lower intracellular levels of glucose, glucose-1-phosphate (G1P), ATP, NAD^+^, fructose, mannose, pyruvate, and lactate, compared with M2b, M2c, and M2d MΦs (Figure 3 and Table S3). ATP is classically associated with M1 MΦ activation, given the increased energy needs of M1 MΦs [19]. Intracellular levels of ATP were higher in M2b, M2c, and M2d MΦ subtypes, relative to M2a MΦs, with M2b MΦs exhibiting the highest ATP levels, suggesting elevated energy demands in these M2 MΦ subtypes compared with M2a MΦs. 

Decreased levels of intracellular glucose are typical markers of increased glycolysis; however, an additional signature of enhanced glycolysis is the increased cellular uptake of glucose [19,32]. When evaluating the significance of the extracellular metabolite concentration patterns, together with the intracellular metabolite profiles (Figure 3 and Figure 4, and Table S3), elevated extracellular levels of glucose suggest that this substrate is not being taken up as much by M2a MΦs compared with the other MΦ subtypes. This reduction in glucose uptake has been previously associated with the generalized M2 MΦ polarization state, along with overall reduced reliance on glycolysis for energy production [19]. In addition to the uptake and utilization of glucose, a branch point of glucose catabolism is glucose-6-phosphate (G6P), which can be directed to the pentose phosphate pathway (PPP) or can be used as a substrate for glycogen synthesis via conversion to G1P and eventual production of uridine diphosphate (UDP)-glucose. 

Glycogen synthesis is associated with an inflammatory MΦ state; however, glycogenolysis (i.e., glycogen degradation), which produces G1P, has also been attributed to the M1 MΦ polarization state. G1P produced from glycogenolysis can be converted to G6P to be used in the oxidative phase of the PPP [33]. This apparently futile cycle of synthesizing and catabolizing glycogen actually plays a valuable role in activating the purinergic P2Y_14_ receptor through UDP-glucose (the key substrate for glycogen synthesis), which mediates the inflammatory phenotype of MΦs [33]. M2a MΦs exhibit significantly (FDR *p* ≤ 0.05) lower intracellular levels of G1P, relative to the other M2 MΦ subtypes, suggesting that M2b, M2c, and M2d MΦs are metabolizing glycogen for G1P production. This would suggest an upregulation of the P2Y_14_ receptor associated with this cyclical glycogen synthesis and degradation process, which could upregulate STAT1 (signal transducer and activator of transcription factor-1) and promote a more M1-like pro-inflammatory polarization state in M2b, M2c, and M2d MΦs compared with M2a MΦs. Further studies are needed to fully explore why M2b, M2c, and M2d MΦs have higher intracellular levels of G1P compared with M2a MΦs.

At the end stage of glycolysis, pyruvate can either be converted to acetyl-coenzyme A (acetyl-CoA) for commitment to the TCA cycle or shunted to lactate production during anaerobic glycolysis to regenerate cytosolic NAD^+^ levels and rapid energy production. Intracellular lactate, a marker of anaerobic glycolysis in MΦs, was found to be elevated in M2b, M2c, and M2d MΦ subtypes compared with M2a MΦs. Taken together and considering the elevated intracellular levels of the TCA cycle intermediate succinate in the M2b, M2c, and M2d MΦ subtypes, these findings suggest that lactate is preferentially being generated as a result of anaerobic glycolysis and that aerobic OXPHOS is reduced in M2b, M2c, and M2d MΦs [26]. Similarly to the metabolite profile signatures of glucose and G1P, elevated lactate levels measured in M2b, M2c, and M2d MΦs suggest that these M2 MΦ subtypes employ anaerobic glycolysis preferentially relative to M2a MΦs, a mechanism that is not commonly associated with a classical M2 MΦ activation state [27]. 

Excess lactate generated as a result of anaerobic glycolysis, which is typically associated with a pro-inflammatory state, is excreted into the extracellular milieu in MΦ s [34]. Extracellular lactate levels were most reduced in M2a MΦs, relative to M2b, M2c, and M2d MΦs, with M2b MΦs exhibiting the highest extracellular lactate levels (Figure 4 and Table S4). Significantly elevated levels of TCA cycle intermediates, including succinate and fumarate, were measured in the intracellular metabolite profiles of M2b, M2c, and M2d MΦ subtypes compared with M2a MΦs (Figure 3 and Table S3), supporting the premise that M2b, M2c, and M2d MΦs rely preferentially on anaerobic glycolysis. However, typically observed with elevated succinate is a decrease in fumarate levels, which would point to an impaired TCA cycle, which does not appear to be the case in the M2 MΦ subtypes examined here. Intracellular fumarate levels display similar trends in all M2 MΦ subtypes, with M2b, M2c, and M2d MΦs exhibiting significantly higher levels of fumarate than M2a MΦs and M2b MΦs having the highest intracellular concentrations (Figure 3 and Table S3). These results suggest that the TCA cycle remains functional at the reaction step catalyzed by succinate dehydrogenase, as indicated by comparable concentrations of succinate and fumarate, which could be fueled by glutaminolysis [35], as discussed further in Section 3.3. The altered metabolite patterns suggesting increased glycolysis in M2b and M2c MΦs are also consistent with their immunometabolic effector functions, which act in pro-inflammation and phagocytic responses, similar to what is observed in M1 MΦs [25]. 

**Figure 10:**
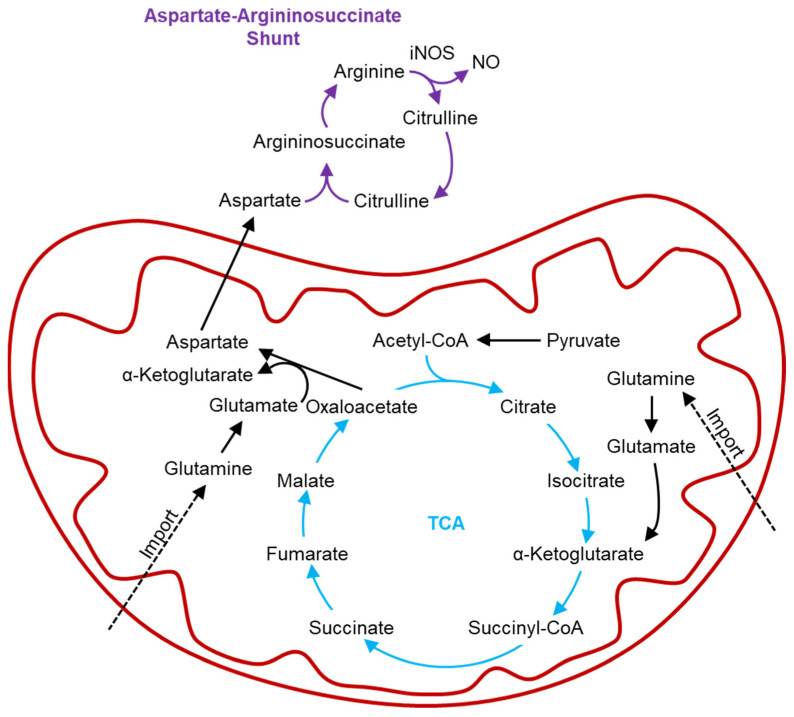
The production of nitric oxide (NO) through the coupling of the TCA cycle (blue) to the aspartate–argininosuccinate shunt (purple). HIF1α stimulates inducible nitric oxide synthesis (iNOS), promoting the conversion of arginine to citrulline and NO. The TCA cycle provides aspartate to produce argininosuccinate, thus maintaining activity of the aspartate– argininosuccinate shunt. Key steps of maintaining the aspartate– argininosuccinate shunt are the import of glutamine into the mitochondria, and its conversion to glutamate. Glutamate can then be used with oxaloacetate to produce aspartate and α-ketoglutarate, which can then be used in the TCA cycle.

Elevated intracellular levels of succinate in M2b, M2c, and M2d MΦs, compared with the levels measured in M2a MΦs, could result from stabilization of the transcription factor HIF-1α (hypoxia inducible factor-1α), which has been reported to drive glycolytic gene expression and initiate glycolysis [26,36], depleting oxygen stores and dampening OXPHOS dependence. Accumulation of HIF-1α is also implicated in driving nitric oxide (NO) synthesis by activating the aspartate–argininosuccinate shunt, which intersects with the TCA cycle, utilizing oxaloacetate to produce aspartate to fuel NO synthesis (Figure 10) [26]. This pathway directly utilizes the amino acids aspartate and citrulline, thus introducing another group of metabolites with levels that differ significantly between M2a, M2b, M2c, and M2d MΦ subtypes. 

### 3.3. Elevated Levels of Amino Acids in M2b, M2c, and M2d MΦs Relative to M2a MΦs

Another subclass of metabolites that distinctly separates M2a MΦs from M2b, M2c, and M2d MΦ subtypes includes amino acids. Specifically, intracellular levels of alanine, arginine, asparagine, aspartate, glutamine, glycine, histidine, isoleucine, leucine, lysine, methionine, phenylalanine, proline, serine, tyrosine, and valine were all lower in M2a MΦs relative to M2b, M2c, and M2d MΦs (Figure 3 and Table S3). Availability of amino acids is crucial for MΦ function and physiological responses as they modulate mTOR signaling, NO production, and the synthesis of small-molecule intermediates with immunomodulatory properties [36]. Many of the amino acids identified with significantly altered concentrations (FDR *p* ≤ 0.05) between M2 MΦ subtypes are relevant to arginine metabolism, the urea cycle, and glutamate and glutamine metabolism. 

Arginine metabolism is a focal point of MΦ polarization research, as the activity of inducible NO synthase (iNOS) and ARG1 enzymes have been associated with M1 and M2 MΦ polarization states, respectively [26]. However, neither iNOS nor ARG1 are exclusively expressed by either pro- or anti-inflammatory myeloid cell subsets, as they can be modulated by different stimuli. ARG1 can be stimulated by elevated levels of lactate and expression of HIF-1α during hypoxia [26], representing a branch of metabolism governed by lactate levels and implicated in the repolarization mechanism of M1 to M2 MΦs. Our metabolomics data further substantiate the delicate balance at play between iNOS and ARG1 activity within M2 MΦ subtypes [24,26]. Our analysis of the metabolite profiles of M2b, M2c, and M2d MΦ subtypes suggests that these M2 MΦ subtypes display an activated, anti-inflammatory M2 phenotype, when assessed by elevated levels of metabolic intermediates associated with ARG1 activity compared with M2a MΦ levels, while still registering metabolite signatures more closely associated with a pro-inflammatory M1-like MΦ phenotype.

When iNOS is active, arginine is converted to citrulline and NO (Figure 10). However, neither NO nor citrulline could be identified and quantified in the current study, thus preventing a detailed analysis of the impact of iNOS activity on the metabolomics profiles of M2 MΦ subtypes investigated herein. Conversely, ARG1 activation leads to an accumulation of ornithine and urea [25], both of which were shown to be in higher concentrations in the intracellular metabolite profiles of the M2b, M2c, and M2d MΦ subtypes (Figure 3 and Table S3) relative to M2a MΦs. Ornithine produced by ARG1 can be utilized for polyamine synthesis, substrates that are integral for cell proliferation and tissue repair, both of which are functions attributed to M2a MΦs [25,26,36]. These metabolomics signatures thus suggest that M2a MΦs utilize ornithine to generate polyamines, which could lead to reduced intracellular levels of ornithine measured in M2a MΦs relative to levels quantified in M2b, M2c, and M2d MΦs. 

### 3.4. Evidence of PPP Activity and Antioxidant Glutathione Metabolism Intersecting with Glycolysis, Glycogenolysis, and Amino Acid Metabolism

Enhanced glycolysis and glycogenolysis are associated with enhanced activity of the oxidative phase of the PPP in MΦs, which has been shown to be primarily supported by the catabolism of glycogen and conversion of G1P to G6P [33]. The oxidative phase of the PPP is important for mediating the inflammatory response of M1 MΦs, being a crucial source of NADPH, which mediates ROS production via NADPH oxidase activity, or provides protection from oxidative stress by providing electron-rich reducing power in the form of NADPH to regenerate reduced glutathione (GSH) following formation of oxidized glutathione (GSSG) during ROS detoxification [33,36]. 

Glutathione metabolism is thus another key pathway associated with MΦ effector functions, which can be initiated by exposure to pathogen-associated molecular patterns (PAMPs) and oxidative stress [26]. GSH levels were found to be significantly lower in M2a MΦs relative to those measured in M2b MΦs (Figure 3 and Table S3). Intracellular NADPH levels revealed similar patterns to those observed for glutathione, though M2a MΦs had the lowest NADPH levels (Figure 3 and Table S3) relative to M2d MΦs, the latter having the highest concentration among all M2 MΦ subtypes. Considering oxidative stress as a whole, the amino acid derivative taurine has been shown to attenuate oxidative stress associated with M1 MΦ function, thereby imparting cytoprotective effects [19]. Intracellular taurine levels were significantly decreased in M2a MΦs relative to levels measured in M2b, M2c, and M2d MΦs (Figure 3 and Table S3). Taken together, these data suggest there is a distinct dynamic of glutathione and taurine utilization by M2a MΦs to mitigate oxidative stress. Overall reduced levels of intracellular taurine in M2a MΦs relative to the other M2 MΦ subtypes indicate this subtype may preferentially utilize taurine to mitigate oxidative stress, especially given the ANOVA analysis (Table S3), indicated marginal statistical differences in the levels of NADPH and GSH in the M2a MΦs compared with the M2b and M2c MΦs.

In each of the pathways discussed, the polar metabolite profiles of M2a MΦs are most distinct from the M2b, M2c, and M2d MΦ subtypes. Despite significant separation of M2a MΦs from the others in 2D and 3D PCA scores plots, significant metabolite level differences between M2b, M2c, and M2d MΦ subtypes became more apparent when examining their metabolome characteristics separately from M2a MΦs. A detailed multivariate statistical analysis of the metabolite patterns of M2b, M2c, and M2d MΦs, excluding those of M2a MΦs, revealed significant metabolome alterations that clearly differentiated M2b, M2c, and M2d MΦ subtypes from each other.

### 3.5. Characteristic Metabolite Profiles Further Differentiate the Distinct Metabolic States of M2b, M2c and M2d MΦs Subtypes

ANOVA analysis of intra- and extracellular metabolite concentrations identified 16 and 17 metabolites, respectively, that contributed significantly to the separate clustering of M2b, M2c, and M2d MΦ subgroups (Figure 6 and Figure 8, and Tables S5 and S6). Metabolites that were significantly (FDR *p* < 0.05) altered in these M2 MΦ subtypes were associated with central metabolic pathways involving lactate fermentation, also known as the endpoint of anaerobic glycolysis, and the TCA cycle. In addition to lactate and TCA cycle intermediates, distinct changes associated with creatine and phosphocreatine metabolism, de novo glycerophospholipid synthesis including the Kennedy pathway, and catabolism of other metabolites were inferred from the distinct intra- and extracellular metabolite profiles measured for M2b, M2c, and M2d MΦs. 

#### 3.5.1. Lactate and TCA Cycle Intermediates Suggest That M2b MΦs Most Closely Adopt an M1 Phenotype Compared with M2c and M2d MΦs

As previously indicated in Section 3.2, the metabolite profiles of M2b, M2c, and M2d MΦs suggest that these M2 MΦ subtypes rely more on rapid energy production via anaerobic glycolysis than usage of the TCA cycle and OXPHOS when compared with M2a MΦs. However, differences in lactate and TCA cycle intermediate levels further distinguished the M2b, M2c, and M2d MΦ subtypes from one another. ANOVA analysis of metabolite concentrations indicated that the polar metabolome of M2b MΦs differs significantly from those of M2c and M2d MΦs, a finding that is consistent with previous reports [23]. 

Differences in extracellular levels of lactate were found to be significant (FDR *p* < 0.05) and clearly differentiated M2b, M2c and M2d MΦs from one another, with M2b MΦ cell cultures having the highest extracellular concentrations of lactate (Figure 8 and Table S6). This observation supports the notion that M2b MΦs exhibit M1-like metabolic characteristics, and their reliance on rapid energy production via anaerobic glycolysis is further substantiated by higher levels of intracellular ATP measured for the M2b MΦs relative to M2c and M2d MΦs and trending lower levels of intracellular NAD^+^ in M2b MΦs relative to M2c MΦs (Figure 6 and Table S5). 

Intracellular levels of metabolites associated with the TCA cycle and identified as significant (FDR *p* < 0.05) discriminators of M2b, M2c, and M2d MΦ subgroups included glutamate, succinate, fumarate, ATP, AMP, and NAD^+^. M2b MΦs exhibited the highest intracellular levels of fumarate, succinate, glutamate, and ATP, and the lowest levels of AMP (Figure 6 and Table S5). Taken together, these metabolite level differences represent unique signatures for each M2 MΦ subtype, and suggest that the M2b subtype is most reliant on rapid energy ATP production via anaerobic glycolysis, as judged on extracellular lactate levels, yet still maintains an active TCA cycle, as based on the comparable levels of succinate and fumarate. However, even though this interpretation is consistent with the observed metabolome patterns, we cannot exclude that these changes reflect metabolic processes at play other than just energetic pathways and canonical flow of intermediates through pathway branchpoints. 

For example, succinate and fumarate play additional roles in MΦ polarization by impacting signaling and promoting adaptive immune responses. As previously mentioned, succinate accumulation is attributed to HIF-1α stabilization, and thus upregulation of the proinflammatory cytokine IL-1β. Succinate can also stimulate IL-1β when it is excreted into the extracellular space by binding the succinate receptor SUCNR1 [35], though differences in extracellular succinate levels were not observed among the M2b, M2c, and M2d MΦs in this study. Fumarate has also been shown to modulate the MΦ epigenome, thereby inducing changes that promote trained immunity, and stimulate proinflammatory cytokine production [35,37]. Typically, these metabolic effector functions are dependent on elevated levels of succinate and fumarate in M1 MΦs, which, in turn, depend on glutaminolysis [35]. Our intracellular metabolite profiles indicate that glutamate levels are higher in M2b MΦs relative to M2c and M2d MΦs (Figure 6 and Table S5), suggesting that, if glutamate is being used to replenish TCA cycle intermediates, then M2b MΦs may be able to increase the levels of α-ketoglutarate (i.e., the keto acid of glutamate) directed to the TCA cycle more efficiently than M2c and M2d MΦs.

#### 3.5.2. Creatine Metabolism Differentiates M2b, M2c, and M2d MΦ Subtypes and Mediates Phagocytosis

Creatine has been shown to shift the polarization state of MΦs towards the M2 phenotype [38]. Though the mechanism by which creatine modulates MΦ polarization states is not well defined, creatine has been shown to suppress the expression of toll-like receptors, including TLR-2, TLR-3, TLR-4, and TLR-7, thereby mitigating a pro-inflammatory response. Creatine has also been shown to mediate chromatin accessibility, thus enhancing a STAT6-mediated transcriptional program towards an M2-like phenotype [38,39]. 

Highest intracellular levels of creatine were observed for M2c MΦs, with M2b and M2d MΦs exhibiting lower but comparable intracellular creatine levels. Extracellular creatine levels presented alternative trends, with M2d MΦs having markedly elevated extracellular creatine levels compared with M2c MΦs (Figure 9). Creatine sequestration from the environment is a key process used to maintain intracellular creatine concentrations within MΦs, and creatine uptake is a primary mechanism used in M2 MΦs to maintain ATP homeostasis during phagocytosis [39]. Interestingly, in this study, M2c MΦs appeared to exhibit the greatest environmental creatine sequestration based on the low levels measured in the spent cell culture media and highest intracellular levels (Figure 9), compared with the intra- and extracellular levels of creatine measured in M2b and M2d MΦs.

Creatine energetics and metabolism contribute to the distinct energy requirements of MΦ phagocytosis, and this pathway also mediates cytokine secretion and proinflammatory responses to stimuli [38,40,41]. Phosphorylation of creatine provides a mean to store energy and helps maintain high ATP:ADP ratios, which are needed to support the MΦ phagocytic function [38,39,41]. In this study, M2b MΦs had the highest levels of intracellular phosphocreatine, although they also had the highest ATP levels, suggesting that phosphocreatine is not being utilized in this subtype to maintain high levels of ATP. In contrast to M2b MΦs, M2c and M2d MΦs had comparable but lower intracellular levels of phosphocreatine and ATP (Figure 6 and Table S5). Given that M2c MΦs specialize in phagocytosis, and the well-defined role played by creatine and phosphocreatine for this MΦ effector function, it could be expected that lower levels of phosphocreatine might be accompanied by higher levels of ATP in M2c MΦs relative to the other M2 MΦ subtypes. While this metabolite pattern was not observed in M2c MΦs, the data suggest the importance of additional metabolic processes besides energetics in mediating M2 MΦ subtype phenotypes and effector functions. 

#### 3.5.3. Phospholipid Synthesis Is Impaired in M2c and M2d MΦ Subtypes

FAO and fatty acid synthesis (FAS) each play dynamic roles in mediating M1 and M2 MΦ polarization and activation states. Lipids taken up by MΦs are processed by lipases, generating free fatty acids (FFAs) and glycerol. FFAs are then transported to the mitochondria for FAO and provide substrates (acetyl-CoA, NADH, and FADH_2_) for the TCA cycle and ATP production via action of the electron transport chain [36]. FAS is an important pathway for prostaglandin biosynthesis in M1 MΦs, and M2 MΦs rely on fatty acid uptake and oxidation for energy production; thus FAS and FAO are regulatory pathways that mediate M1 and M2 polarization states [19]. 

Altered levels of metabolites involved in de novo glycerophospholipid synthesis and degradation were found to be significant (FDR *p* < 0.05) discriminators of the M2b, M2c, and M2d MΦ subtypes. Intracellular levels of choline, the substrate for phosphatidylcholine (PC) synthesis, were highest in M2c MΦs, with M2d MΦs exhibiting the next highest levels, and M2b MΦs having the lowest intracellular levels of choline (Figure 9). Extracellular choline levels were highest in M2d MΦs, and lowest in M2c MΦs, with the M2b MΦs displaying a large variability in choline profiles (Figure 8 and Figure 9). Intracellular levels of phosphoethanolamine (PEtn), the product of the committed step to phosphatidylethanolamine (PE) synthesis, were highest in M2c and M2d MΦ subtypes, with M2b MΦs having the lowest intracellular levels (Figure 6 and Table S5). Lastly, intracellular levels of glycerophosphocholine, an intermediate of PC catabolism that produces choline, were most elevated in M2d MΦs, and lowest in M2b MΦs (Figure 6 and Table S5). Taken together and factoring in the levels of ATP and AMP measured, these data suggest that M2c and M2d MΦs may not be committing choline and ethanolamine to phospholipid synthesis to the same extent as M2b MΦs, as revealed by high intracellular levels of choline and PEtn, and reduced levels of ATP in M2c MΦs and M2d MΦs compared with the levels measured in M2b MΦs. 

The first step of either PC or PE synthesis requires ATP, whose intracellular concentrations were lower in M2c MΦs and M2d MΦs, relative to M2b MΦs (Figure 6 and Table S5). As previously stated, it could be expected that an M2 MΦ would downregulate FAS and upregulate FAO, which appears to be the case regarding phospholipid synthesis for M2c MΦs and M2d MΦs. Catabolism of PC was indicated by higher intracellular levels of glycerophosphocholine in M2c MΦs and M2d MΦs relative to M2b MΦs (Figure 6 and Table S5). The metabolic signatures of M2c MΦ and M2d MΦ subtypes suggest increased catabolism of PC to enhance choline production. However, based on the very low extracellular levels of choline measured in M2c MΦs compared with the higher extracellular levels observed for M2d MΦs (Figure 8 and Table S6), these metabolite patterns suggest a reduced uptake of choline in M2d MΦs and the possibility that choline may be utilized for an function other than only contributing to the Kennedy pathway. 

Recent literature has suggested that choline uptake at the site of inflammation serves multiple biological functions. Among these, choline uptake has been shown to mediate IL-1β and interleukin-18 (IL-18) secretion, which are cytokines classically associated with M1 and M2b MΦs [42,43]. However, in the absence of additional cytokines associated with M1 MΦs, IL-18 has been reported to stimulate the secretion of IL-13 and IL-4, which are canonical M2 MΦ cytokines [43,44]. Thus, differential intra- and extracellular levels of choline may be reporting on specific processes by which M2c MΦs are uniquely distinguished from M2b and M2d MΦ subtypes, and present an opportunity for future studies. 

## 4. Materials and Methods

### 4.1. Isolation of Human Monocytes

Heparinized venous whole blood was obtained from four healthy adult donors with Montana State University Institutional Review Board approval (#VC100118) and written informed consent. Isolation of peripheral blood mononuclear cells (PBMCs) from whole donor blood by density gradient centrifugation and magnetic-activated cell sorting (MACS), respectively, was performed, as previously described [19].

### 4.2. Differentiation and Activation of Human Monocyte-Derived MΦs

MΦs were differentiated from isolated primary human monocytes and activated following similar protocols to those reported previously [19]. M2a MΦs were generated by stimulation with 20 ng/mL of recombinant IL-4 (PeproTech Inc., Cranbury, NJ, USA) for 72 h, as reported in our previous study [19]. M2b and M2c MΦs were stimulated for 72 h (on culture days 7–9) with 50 ng/mL of recombinant human IL-1β (PeproTech Inc., Cranbury, NJ, USA) and 50 ng/mL recombinant human IL-10 (PeproTech Inc., Cranbury, NJ, USA), respectively. M2d MΦs were stimulated throughout the entire differentiation process (on culture days 0–9) using 50 ng/mL recombinant human IL-6 (PeproTech Inc., Cranbury, NJ, USA) [45].

### 4.3. Antibodies and Fluorescence-Activated Cell Sorting (FACS) Analysis

Following differentiation or stimulation, M2b, M2c, and M2d MΦs were characterized using flow cytometry (Figure S1). Mouse anti-human monoclonal antibodies used in the flow cytometry analysis included CD80 (clone 19.2), CD163 (clone GHI/61), and CD206 (clone L307.4), purchased from BD Biosciences (San Jose, CA, USA). Relevant isotype-matched control antibodies were used in all FACS experiments.

For FACS analysis, cells were labeled with concentrations of antibodies according to the manufacturer’s recommendations at 4 °C for 1 h followed by washing with FACS buffer (1× phosphate-buffered saline (PBS), 2% (*v*/*v*) fetal bovine serum (FBS), and 0.1% (*w*/*v*) sodium azide). A LSR Fortessa flow cytometer (BD Biosciences) was employed and FACS data were analyzed using the FlowJo 10.7.1 software (Ashland, OR, USA). Normalized mean fluorescence intensity (MFI) values were determined, as described in [19].

Based on flow cytometry analysis, the average number of MΦs harvested from each T25 cm^2^ tissue culture flask was determined to be 1.91 × 10^7^ ± 2.37 × 10^6^ (n = 3) cells.

### 4.4. Extraction of Intra- and Extracellular Polar Metabolites for ^1^H NMR Analysis

Extraction of intra- and extracellular metabolites was conducted, as described in our previous study [19]. In brief, sham media controls consisted of supplemented cell culture media without MΦs present, which were handled precisely the same as experimental MΦ cell cultures regarding incubation temperature, duration, and harvest. In contrast, our experimental MΦ cell cultures consisted of supplemented cell culture media with MΦs present prior to spent MΦ cell culture media collection. Aliquots of sham and spent MΦ cell culture medium were filtered using pre-washed Amicon ultra 3-kDa molecular weight cut-off 0.5 mL centrifugal filters (Millipore Sigma, Burlington, MA, USA). Intracellular aqueous metabolite extractions (i.e., extractions of water-soluble small-molecule metabolites) were performed using a combination of 50% aqueous methanol and chloroform [19]. Cell lysis was achieved using a FastPrep-24 5G homogenizer (MP Biomedicals, Santa Ana, CA, USA), followed by partitioning and separation of aqueous and nonpolar phases. Both aqueous intra- and filtered extracellular metabolite extracts were vacuum-dried overnight, with no heat, and stored at −80 °C. Immediately prior to data acquisition, dried metabolite extracts were resuspended in 600 µL of NMR buffer consisting of 25 mM sodium phosphate, 0.4 mM imidazole, and 0.25 mM DSS (4,4-dimethyl-4-silapentane-1-sulfonic acid) in 90% H_2_O/10% D_2_O (pH 7.0), as described previously [19].

### 4.5. ^1^H NMR Spectroscopy

Acquisition of NMR spectra was conducted similarly to that described in [19]. In brief, ^1^H NMR spectra were acquired at 298 K (25 °C) on a 600 MHz (^1^H Larmor frequency) AVANCE III solution NMR spectrometer (Bruker, Billerica, MA, USA), equipped with a 5 mm liquid-helium-cooled, triple-resonance, three-channel inverse (TCI) cryoprobe. One-dimensional (1D) ^1^H NMR spectra were recorded using a ‘zgesgp’ Bruker-supplied pulse sequence with 256 scans per sample and a 9615.38 Hz ^1^H spectral window. Free induction decays (FIDs) were acquired using a dwell time interval of 52 μsec and 32K data points, amounting to a 1.7 sec acquisition time. With a D1 delay time set to 1 s, the total relaxation recovery period between scans thus amounted to 2.7 s.

### 4.6. NMR Data Processing, Metabolite Identification, and Quantitation

Processing of NMR spectra included application of a line-broadening function of 0.3 Hz, a ‘qfil’ polynomial function of 0.2 ppm spectral width to remove the water signal, and phase correction, using the Bruker Topspin v. 3.6 software (Bruker Scientific LLC, Billerica, MA, USA). Chemical shift referencing was accomplished by setting the upfield signal of DSS to 0.0 ppm. The NMR signal of imidazole was used to correct for small chemical shift variations arising from slight pH deviations. 

Further processing and analysis of 1D ^1^H NMR spectra were conducted using Chenomx NMR Suite software (version 8.4; Chenomx Inc., Edmonton, AB, Canada), as previously described [19,46]. In brief, metabolites were identified and quantified by matching characteristic chemical shifts, spectral patterns, and signal intensities to those of small-molecule reference spectra present in the Chenomx spectral database of small-molecule metabolites for 600 MHz (^1^H Larmor frequency) NMR spectrometers. 

Metabolite IDs were verified, as needed, using two-dimensional (2D) ^1^H-^1^H total correlation spectroscopy (TOCSY) experiments, as reported in [19], or spiking of pure compounds.

Quantitative intra- and extracellular metabolite profiles were exported from the Chenomx software as μM concentrations, with metabolic profiles for blank NMR buffer and sham controls generated in parallel with the M2 MΦ cell culture experiments. Blank NMR buffer control spectra were collected with an aliquot of NMR buffer that was not subject to any further manipulations, and sham extracts were collected on media, subject to the same experimental processes as the macrophage cultures, except the addition of any macrophages. Concentrations of small molecules measured in blank NMR buffer control and sham extracts were subtracted from the metabolite profiles obtained for the intracellular and extracellular M2 MΦ samples, respectively.

### 4.7. Determination of Intracellular Protein Concentrations and Sample Biomass Normalization

Intracellular protein concentrations, obtained from aliquots of cellular lysates that were stored at −80 °C, were established using a Pierce bicinchoninic acid (BCA) protein assay kit (ThermoFisher Scientific, Waltham, MA, USA), as specified by the manufacturer-provided user guide and as described previously [19].

Prior to univariate and multivariate statistical analysis using MetaboAnalyst [47,48], intra- and extracellular metabolite concentrations were converted from μM to nmol by accounting for NMR buffer volume, and normalized to protein content (biomass) to yield final units of nmol/mg protein.

### 4.8. Univariate and Multivariate Statistical Analysis

For statistical analysis, metabolite concentration data were further normalized through log-transformation and auto-scaling (i.e., mean centered and divided by standard deviation) to ensure a Gaussian data distribution. These datasets were then subject to univariate and multivariate statistical analyses, which included analysis of variance (ANOVA), unsupervised principal component analysis (PCA), supervised partial least square-discriminant analysis (PLS-DA), and hierarchical clustering analysis (HCA). 

For univariate and parametric analysis, a false discovery rate (FDR) was set to a *p*-value threshold of <0.05 for ANOVA. A Tukey’s post-hoc analysis was performed on all metabolites determined to be statistically significant (*p* < 0.05), based on concentration differences and one-way ANOVA analyses [19,49]. HCA was conducted in MetaboAnalyst [47,48] using a Euclidean distance measure and Ward clustering algorithm.

The extent of group separations and evaluation of metabolites contributing to the different clustering of the M2 MΦ subgroups in the 2D and 3D PCA scores plots were assessed using metabolites of importance plots associated with each principal component, which were generated using the caret and MixOmics software packages in R (version 3.45) [50,51]. 

Validation and robustness of the clustering of the distinct M2 MΦ subgroups, as observed in the 2D and 3D PLS-DA scores plots, were assessed using a leave-one-out test for predictive variability (Q^2^) and predicted residual sum of squared (PRESS) error (R^2^), and the (B/W) permutation test function (#tests = 2000) of MetaboAnalyst [47]. Additional PLS-DA validation metrics, including classification error rate (CER) and area under the receiver operating characteristic curve (AUROC) analysis, were obtained using the ‘MixOmics’ package in R [49,51].

Variable importance in projection (VIP) plots were used to assess the importance of each metabolite (and associated level change) in the validated PLS-DA models. Metabolites with VIP scores > 1.2 were considered to be significant discriminators of the different M2 MΦ subtypes [49].

## 5. Conclusions

The present study highlights distinct metabolic differences in M2 MΦ subtypes, further supporting the notion that there are unique metabolite patterns that modulate MΦ polarization into distinct M2a, M2b, M2c and M2d MΦ subtypes. Findings from this study provide further evidence that MΦ polarization and activation states exist on a continuum rather than a well delineated, biphasic M1/M2 classification system as originally theorized [52]. Most of the discriminatory metabolites identified here between M2a MΦs from the M2b, M2c and M2d MΦ subtypes indicate that M2b, M2c and M2d MΦs adopt more of an M1-like phenotype with respect to their polar metabolome profiles, as highlighted by their distinct signatures of enhanced glycolysis and regulation of TCA cycle intermediates compared with those observed for M2a MΦs. Differences in arginine metabolism also suggest a trend toward an M1-pro-inflammatory state for M2b, M2c, and M2d MΦs, given their elevated intracellular levels of ornithine, and alterations in arginine, glutamine, glutamate, and other amino acids (alanine, asparagine, aspartate, glycine, histidine, isoleucine, leucine, lysine, methionine, phenylalanine, proline, serine, tyrosine, and valine) and glutathione levels compared with M2a MΦs.

The widespread differences observed between M2a MΦs and M2b, M2c, and M2d MΦs may be due in part to M2a MΦs expressing CCL17, which explicitly inhibits activation towards an M1, or pro-inflammatory, state and is not expressed in the other M2 MΦ subtypes [14,53]. Our metabolome profiles correlate with MΦ specialized functions, such as phagocytosis, and are consistent with other published studies [23]. Though further targeted investigations are needed to draw specific conclusions regarding the similarities of M2 MΦ subtypes to M1 MΦs, this study supports the notion that, given MΦs’ needs to respond to numerous and highly variable stimuli, M2 MΦ subtypes adapt their metabolism and polarization states for optimal effector functions, giving rise to unique characteristics that encompass a physiological continuum between two extremes, as defined by a binary pro-inflammatory M1 and anti-inflammatory M2 MΦ paradigm [52]. 

Fewer metabolic differences were observed when comparing M2b, M2c, and M2d MΦs to each other, although metabolic signatures regarding glucose metabolism, TCA cycle intermediates, creatine and phosphocreatine metabolism, and the Kennedy pathway differentiated these subtypes of M2 MΦs. M2b MΦs are markedly characterized by TNF-α expression, which is not distinctly a pro-inflammatory cytokine, but, rather, mediates paradoxical anti-inflammatory and immune-modulatory effects [14]. TNF-α is secreted primarily by M1 MΦs and M2b MΦs, but additional markers help distinguish between these two polarization states [14]. M2b MΦs regulate the breadth and depth of inflammatory responses, secreting both pro- (IL-1β, IL-6, and TNF-α) and anti-inflammatory cytokines (IL-10) [14]. The dichotomy of expressing similar cytokines and signaling molecules as M1 MΦs results in M2b MΦs being either protective and being considered part of the canonical class of M2 MΦs, or pathogenic when M2b MΦs adopt more of an M1-like phenotype and are improperly regulated. The variability in metabolite patterns observed in M2b MΦs in our study might thus be attributed to the wide range of effector functions mediating the anti-inflammatory responses of M2b MΦs, and may reflect their wider range of metabolic adaptations compared with those employed by M2a, M2c, and M2d MΦs. 

In summary, this work brings new knowledge about characteristics metabolite patterns of primary human M2 MΦ subtypes that are associated with their differential effector functions. This study expands upon previous work that sought to define immunometabolic functions mediating M2 MΦ polarization and differentiation into M2a, M2b, M2c, and M2d MΦ subtypes [23,29]. The present study provides further evidence that metabolism is an intrinsic element of primary human MΦ functions, and much remains to be better understood regarding the cross talk between metabolism, metabolic networks, MΦ phenotypes, and the diverse immune effector functions of distinct M2 MΦ subtypes.

## Data Availability

The data presented within this study are available upon reasonable request from the corresponding author.

## Supplementary Materials

The following supporting information can be downloaded at: https://www.mdpi.com/article/10.3390/ijms25042407/s1.
