# Supplementary material for: Primary Human M2 Macrophage Subtypes Are Distinguishable by Aqueous Metabolite Profiles
Source: Int J Mol Sci. 2024 Feb 18;25(4):2407. doi: 10.3390/ijms25042407 (PMC10889249; doi:10.3390/ijms25042407)
Supplement: Supplementary file 1 [file ijms-25-02407-s001.zip › ijms-2829165-supplementary.pdf]

Article

Primary human M2 macrophage subtypes are distinguishable by aqueous metabolite profiles

Amanda L. Fuchs [†,‡], Stephanann M. Costello [‡], Sage M. Schiller, Brian P. Tripet, and Valérie Copié *

Department of Chemistry and Biochemistry, Montana State University, Bozeman, MT 59717, USA; amanda.fuchs@nih.gov (A.L.F.); stephanann.costello@montana.edu (S.M.C.); sageschiller2@gmail.com (S.M.S.); brian.tripet@montana.edu (B.P.T.)

* Correspondence: vcopie@montana.edu; Tel.: +1-406-994-7244

† Current address: National Institutes of Health, National Human Genome Research Institute, Metabolic Medicine Branch, Metabolism, Infection, and Immunity Section, Bethesda, MD 20892, USA.

‡ These authors contributed equally

Supplementary material included herein consists of the following:

(A) Supplementary Figures:

Supplementary Figure S1: Phenotypic characteristics of primary human monocyte-derived macrophages (MΦs).

Supplementary Figure S2: Representative 1D ^{1}H NMR spectrum of extracellular metabolite extracts obtained from M2 MΦ subtypes.

Supplementary Figure S3. 2D and 3D PLS-DA scores plots analysis of M2a, M2b, M2c, and M2d MΦ subtype intra- (A-B) and extracellular (C-D) polar metabolite profiles.

Supplementary Figure S4. Validation metrics associated with the PLS-DA modeling of the intracellular metabolite profiles of M2a, M2b, M2c and M2d MΦ subtypes.

Supplementary Figure S5. Validation metrics associated with the PLS-DA modeling of the extracellular metabolite profiles of M2a, M2b, M2c and M2d MΦ subtypes.

Supplementary Figure S6. Variable Importance in Projection (VIP) score plots associated with the PLS-DA analysis of the intracellular and extracellular metabolite profiles of M2a, M2b, M2c, M2d MΦs.

Supplementary Figure S7. Hierarchical clustering analysis (HCA) and heatmap visualizations of M2a (blue), M2b (red), M2c (green), and M2d (yellow) MΦ subtype **(A)** intra- and **(B)** extracellular polar metabolite profiles.

(B) Supplementary Tables:

Table S1. Polar metabolites identified in intracellular metabolite extracts of M2 MΦ subtypes.

Table S2. Polar metabolites identified in extracellular metabolite extracts of M2 MΦ subtypes.

Table S3. One-way parametric ANOVA analysis of intracellular M2a, M2b, M2c, and M2d MΦ metabolite levels with Tukey's post-hoc analysis.

Table S4. One-way parametric ANOVA analysis of extracellular M2a, M2b, M2c, and M2d MΦ metabolite levels with Tukey's post-hoc analysis.

Table S5. One-way parametric ANOVA analysis of intracellular M2b, M2c, and M2d MΦ metabolite levels with Tukey's post-hoc analysis.

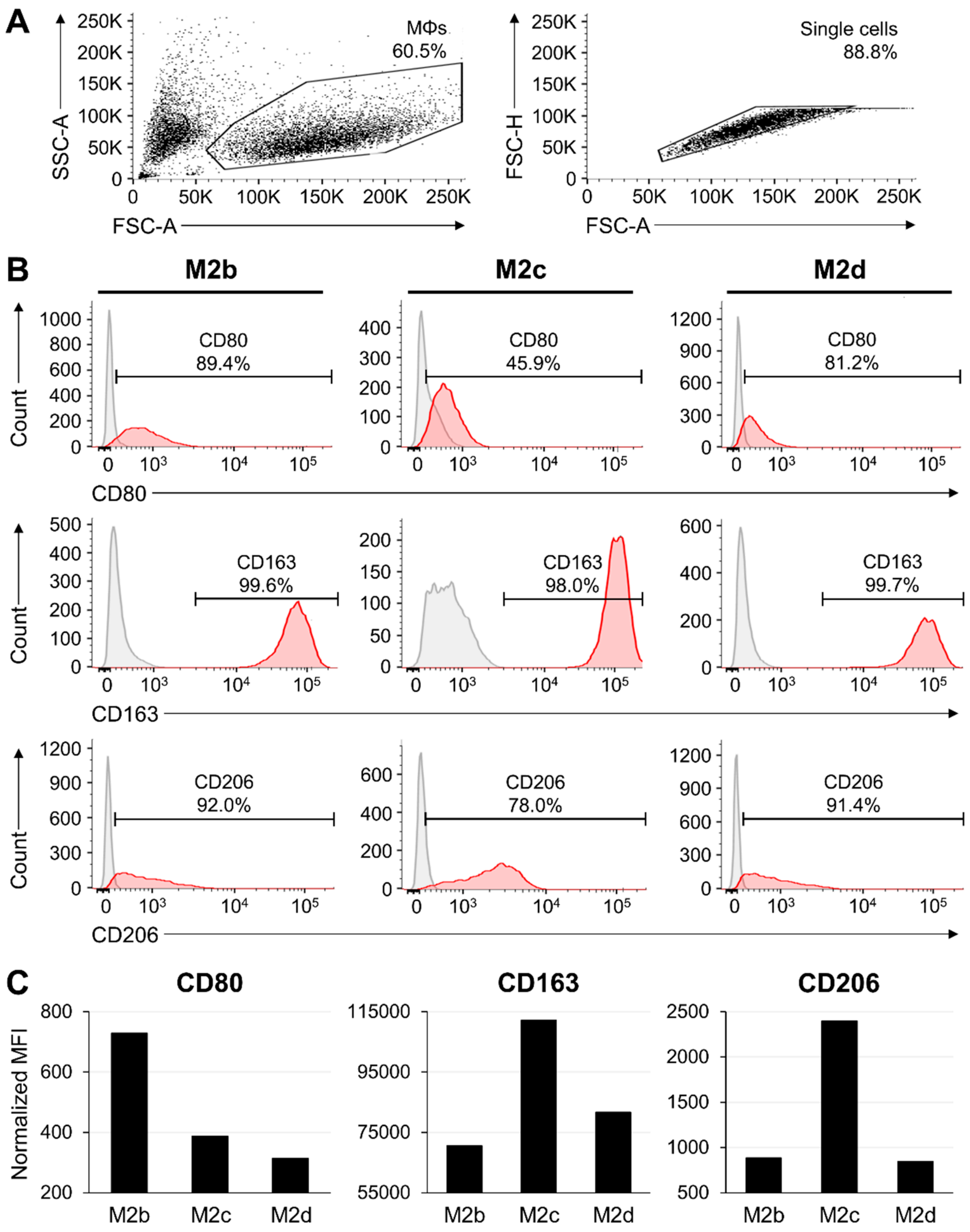

Figure S1. Phenotypic characteristics of primary human monocyte-derived macrophages (MΦs). **(A)** Gating strategy for FACS analysis of MΦs; **(B)** Histograms of CD80, CD163, and CD206 expression (red) by M2b, M2c, and M2d MΦs along with appropriate isotype controls (gray); **(C)** Normalized mean fluorescence intensity (MFI) of CD80, CD163, and CD206 expression by M2b, M2c, and M2d MΦs.

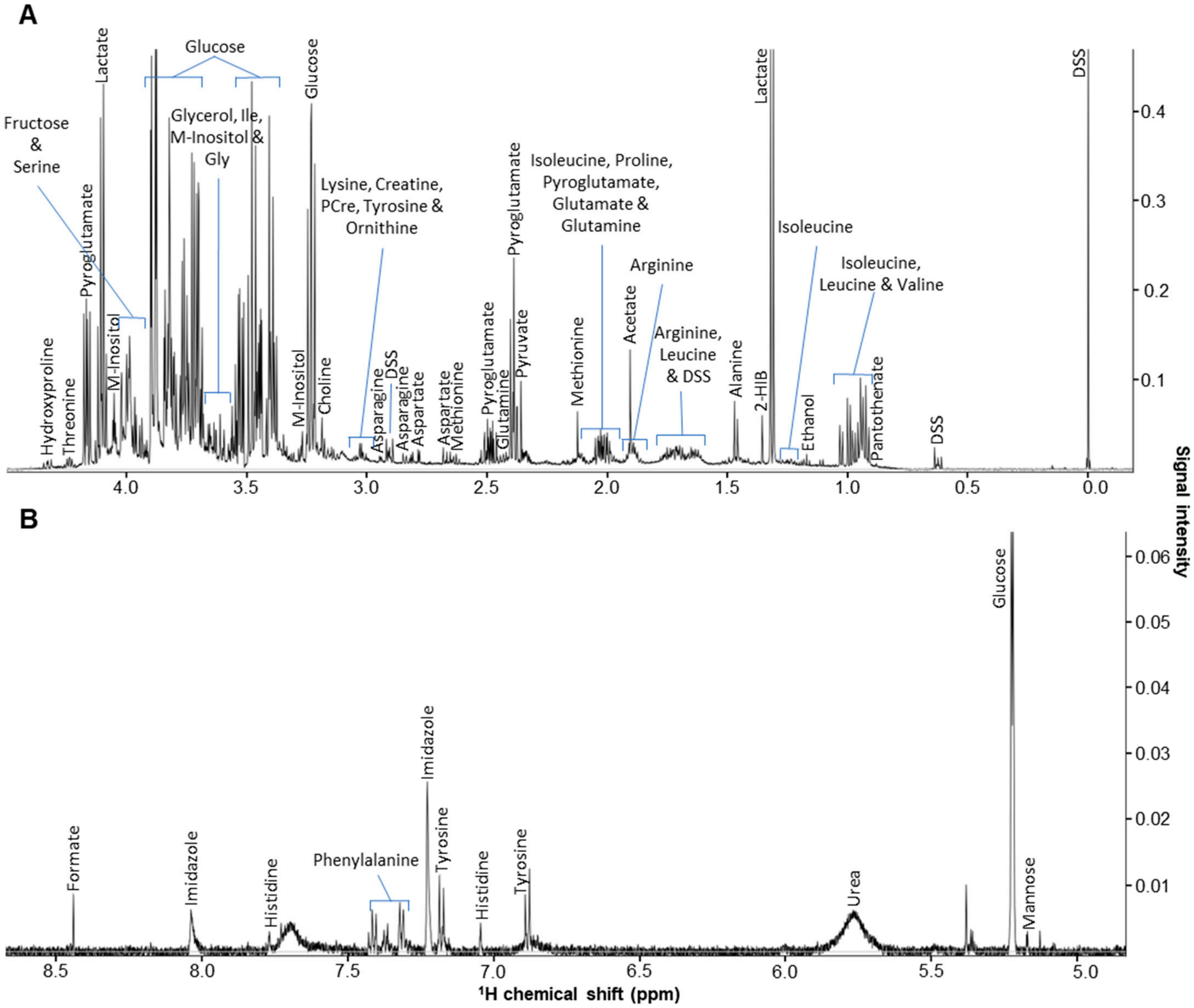

Figure S2. Representative 1D ^{1}H NMR spectrum of extracellular metabolite extracts obtained from M2 MΦ subtypes. ^{1}H chemical shift regions corresponding to the **(A)** 0.0 to ~4.5 ppm and **(B)** 5.0 to ~9 ppm spectral regions are depicted. Abbreviations denote: DSS, 4,4-dimethyl-4-silapentane-1-sulfonic acid.

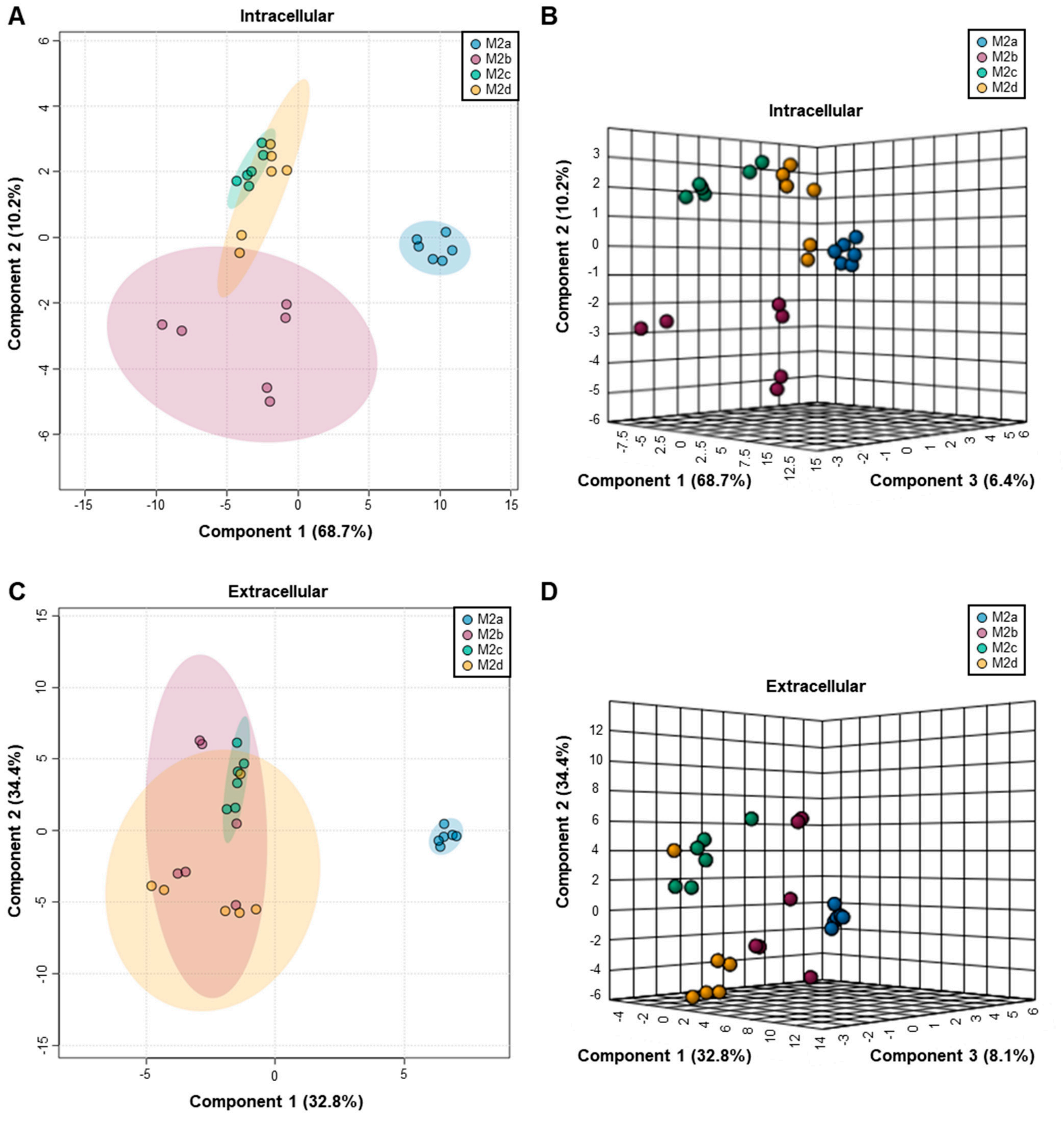

Figure S3. 2D and 3D PLS-DA scores plots analysis of M2a, M2b, M2c, and M2d MΦ subtype intra- (A-B) and extracellular (C-D) polar metabolite profiles. Shaded ellipses indicate corresponding 95% confidence intervals.

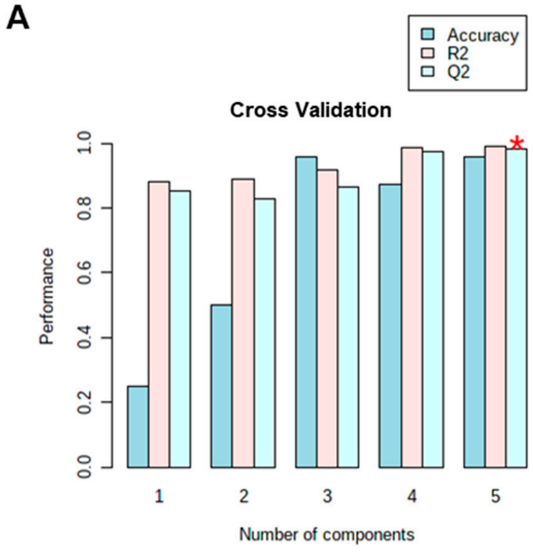

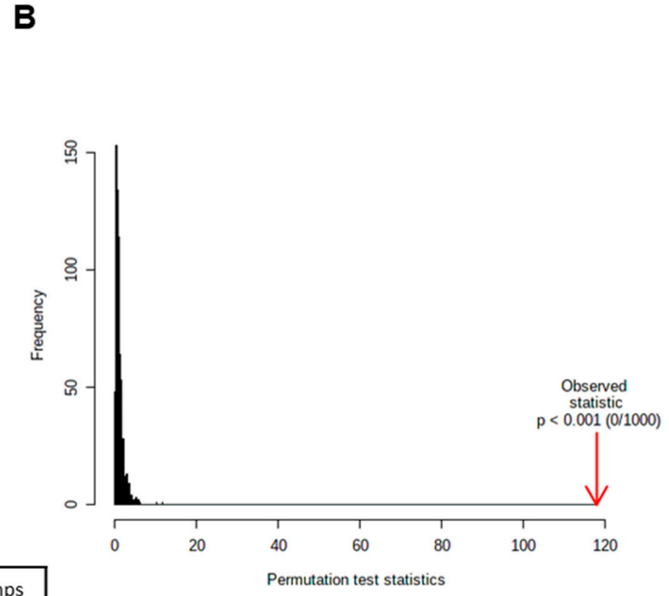

Measure	1 comps	2 comps	3 comps	4 comps	5 comps
Accuracy	0.250	0.500	0.958	0.875	0.958
R^2	0.882	0.889	0.916	0.987	0.992
Q^2	0.852	0.827	0.863	0.975	0.981

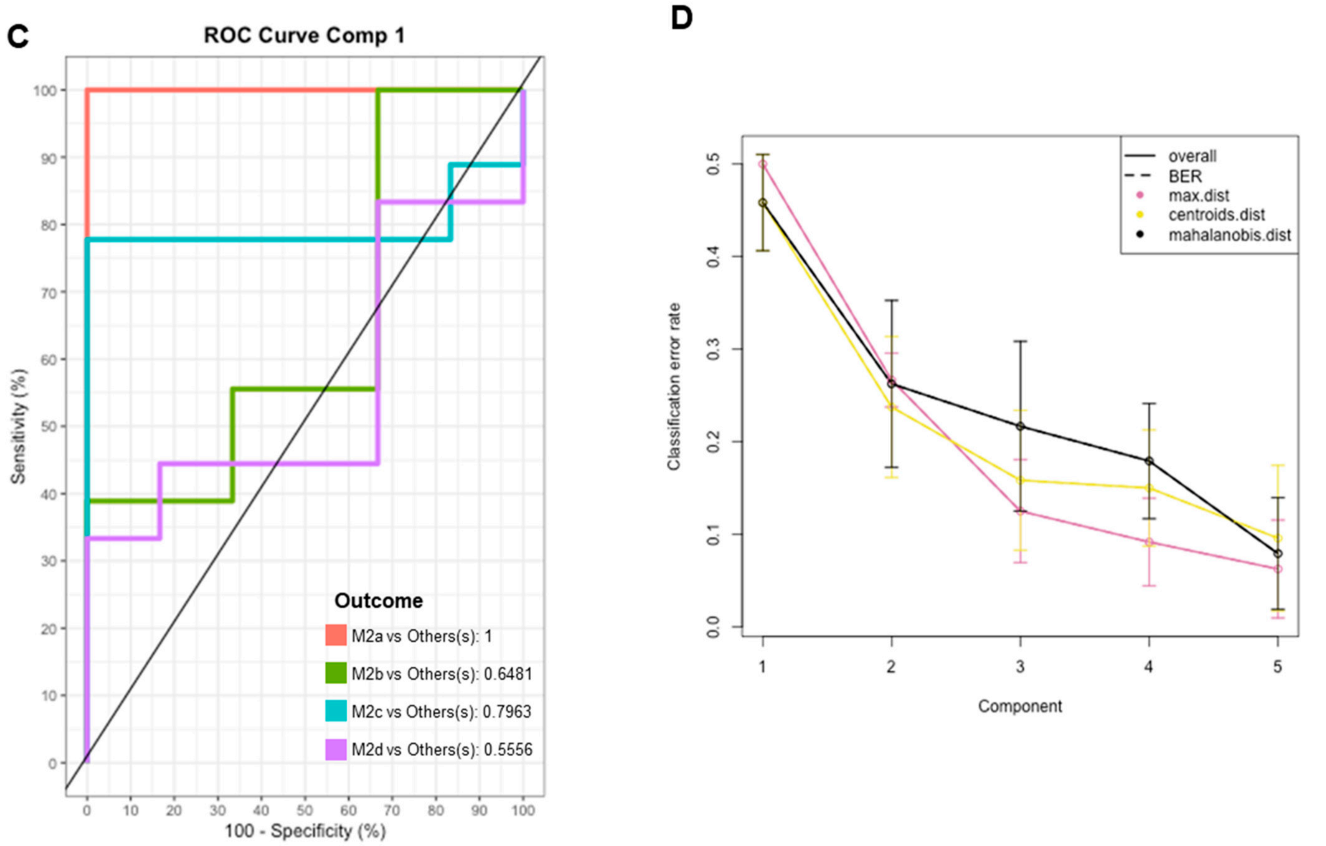

Figure S4. Validation metrics associated with the PLS-DA modeling of the intracellular metabolite profiles of M2a, M2b, M2c and M2d MΦ subtypes. (A) Leave-one-out cross validation (LOOCV) plot demonstrated model

significance with an accuracy score of 0.958, an R2 of 0.992, and Q2 of 0.981, when taking into account the first 5 components of the PLS-DA model. The accuracy score (0.958), R2 (0.916), and Q2 (0.863) scores also demonstrated model significance by the 3rd component. The prediction accuracy (B) permutation test (n = 1000 tests) yielded a significant p-values < 0.001, attesting to the validity and prediction accuracy of the PLS-DA model, demonstrating high model accuracy and specificity for the separate classification of M2 MΦ subtypes. (C) The area under receiver operating characteristic (ROC) curves yielded an AUC value of 1, 0.64, 0.80, and 0.55 for the comparison of M2a, M2b, M2c and M2d MΦ subtypes, respectively. (E) Classification error rate (CER) yielded an overall classification error rate of ~25% for component 3 and less than 25% for component 4 (and beyond) of the PLS-DA model.

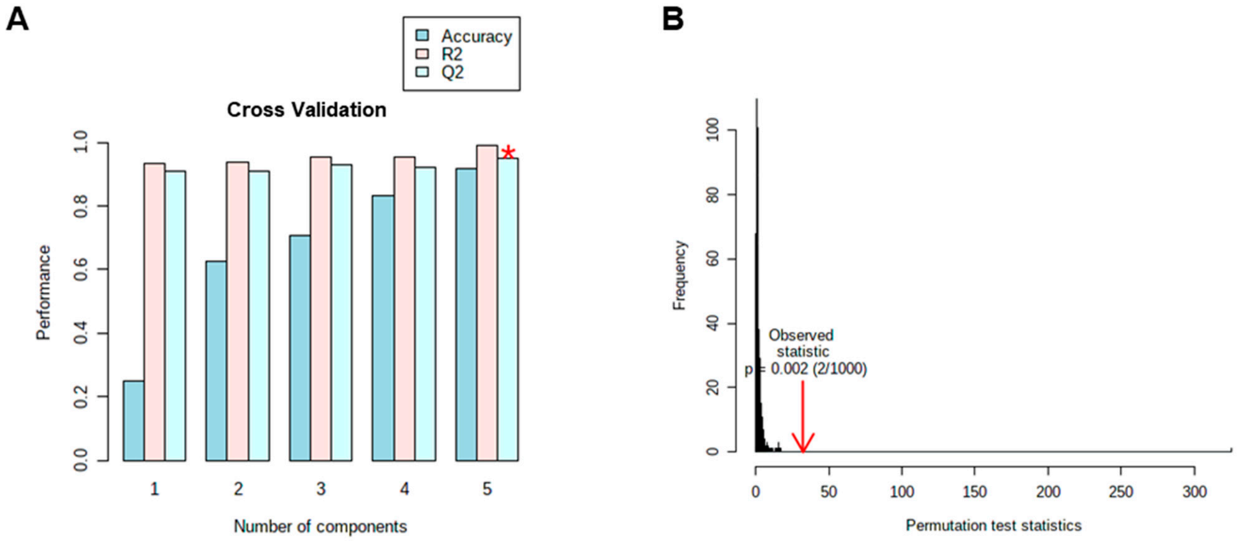

Measure	1 comps	2 comps	3 comps	4 comps	5 comps
Accuracy	0.250	0.625	0.708	0.833	0.917
R^2	0.935	0.939	0.953	0.954	0.990
Q^2	0.909	0.909	0.929	0.922	0.952

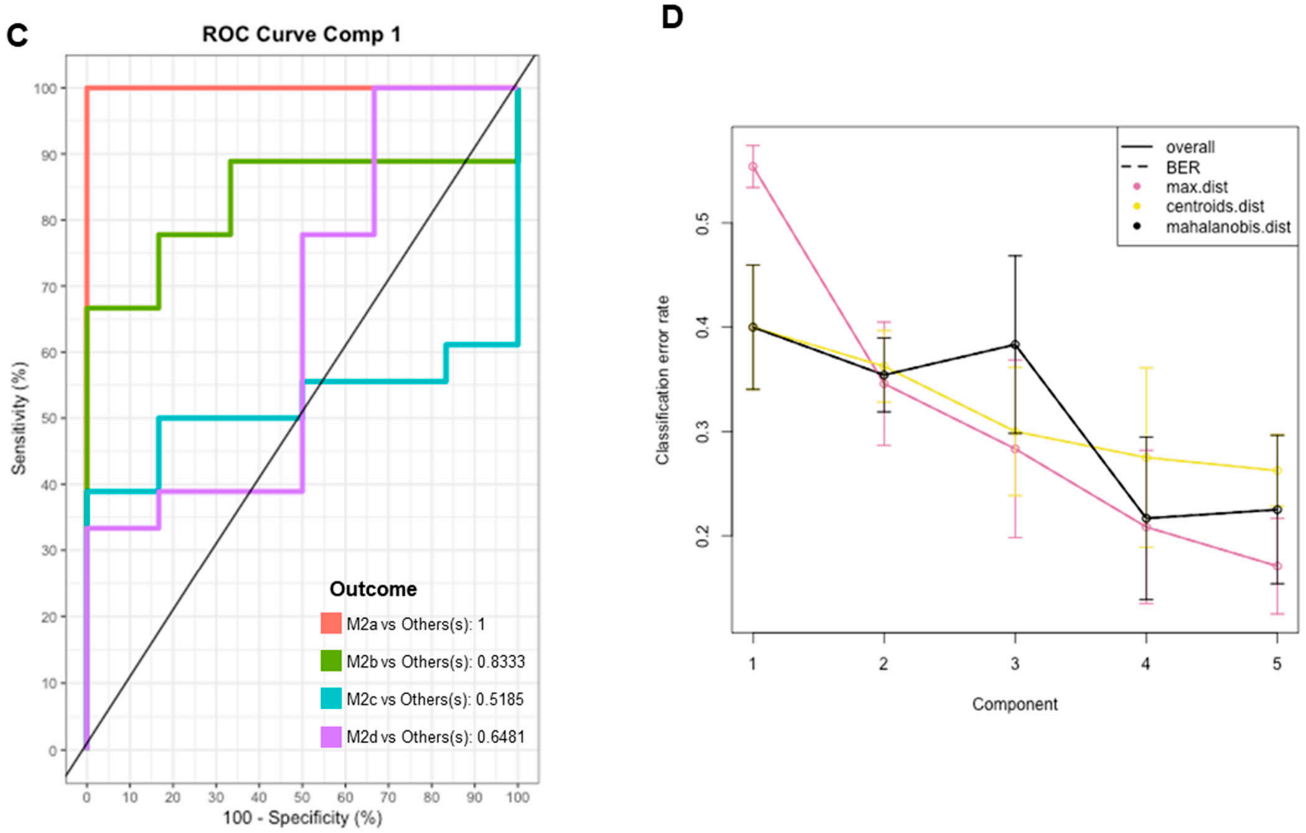

Figure S5. Validation metrics associated with the PLS-DA modeling of the extracellular metabolite profiles of M2a, M2b, M2c and M2d MΦ subtypes. (A) Leave-one-out cross validation (LOOCV) plot demonstrated model

significance with an accuracy score of 0.917, an R^2 of 0.990, and Q^2 of 0.952, when taking into account the first 5 components of the PLS-DA model. The accuracy score (0.708), R^2 (0.953), and Q^2 (0.929) scores also demonstrated model significance by the 3rd component. The prediction accuracy (B) permutation test (n = 1000 tests) yielded a significant p-values < 0.002, attesting to the validity and prediction accuracy of the PLS-DA model, demonstrating high model accuracy and specificity for the separate classification of M2 MΦ subtypes. (C) The area under receiver operating characteristic (ROC) curves yielded an AUC value of 1, 0.83, 0.52, and 0.64 for the comparison of M2a, M2b, M2c and M2d MΦ subtypes, respectively. (E) Classification error rate (CER) yielded an overall classification error rate of ~40% for component 3 and less than 30% for component 4 (and beyond) of the PLS-DA model.

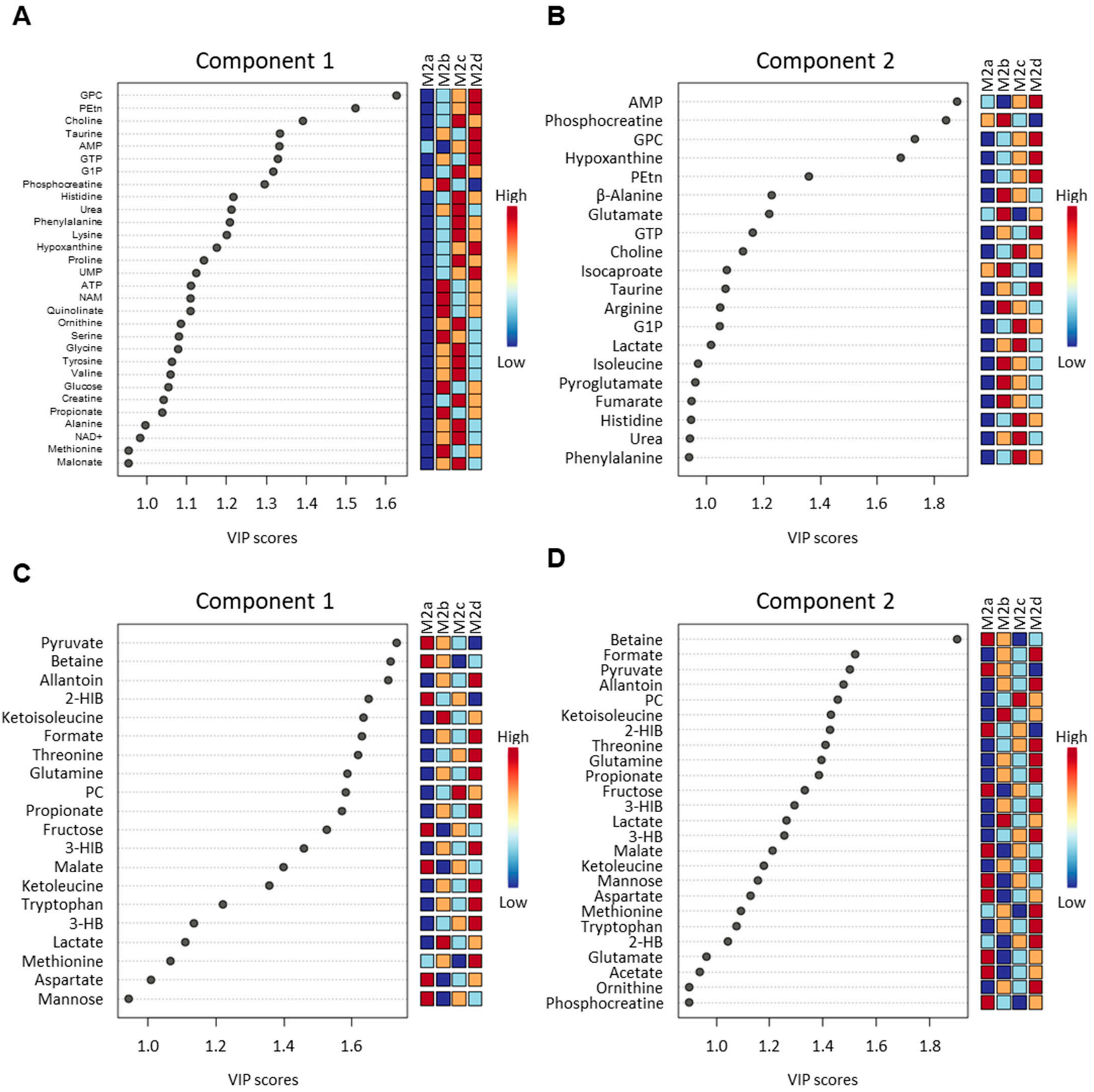

Figure S6. Variable Importance in Projection (VIP) score plots associated with the PLS-DA analysis for intracellular and extracellular metabolite profiles of M2a, M2b, M2c, M2d MΦs. Panels correspond to the intracellular (A-B) and extracellular (C-D) PLS-DAs, indicating which metabolites contribute most to the separation observed along component 1, and component 2 of both the intra- and extracellular PLS-Das (Figure S3). The heatmap gradient indicates the metabolite level is relatively high or low in the corresponding M2 MΦ subtype. Abbreviations: GPC, glycerophosphocholine; PEtn, phosphatidyl ethanolamine; AMP, adenosine monophosphate; GTP, guanosine triphosphate; G1P, glucose-1-phosphate; UMP, uridine monophosphate; ATP, adenosine triphosphate; NAM, niacinamide; NAD+, nicotinamide adenine dinucleotide; 3-HIB, 3-hydroxyisobutyrate; PC, phosphocholine; 3-HIB, 3-hydroxyisobutyrate; 3-HB, 3-hydroxybutyrate; 2-HIB, 2-hydroxyisobutyrate; and 2-HB, 2-hydroxybutyrate.

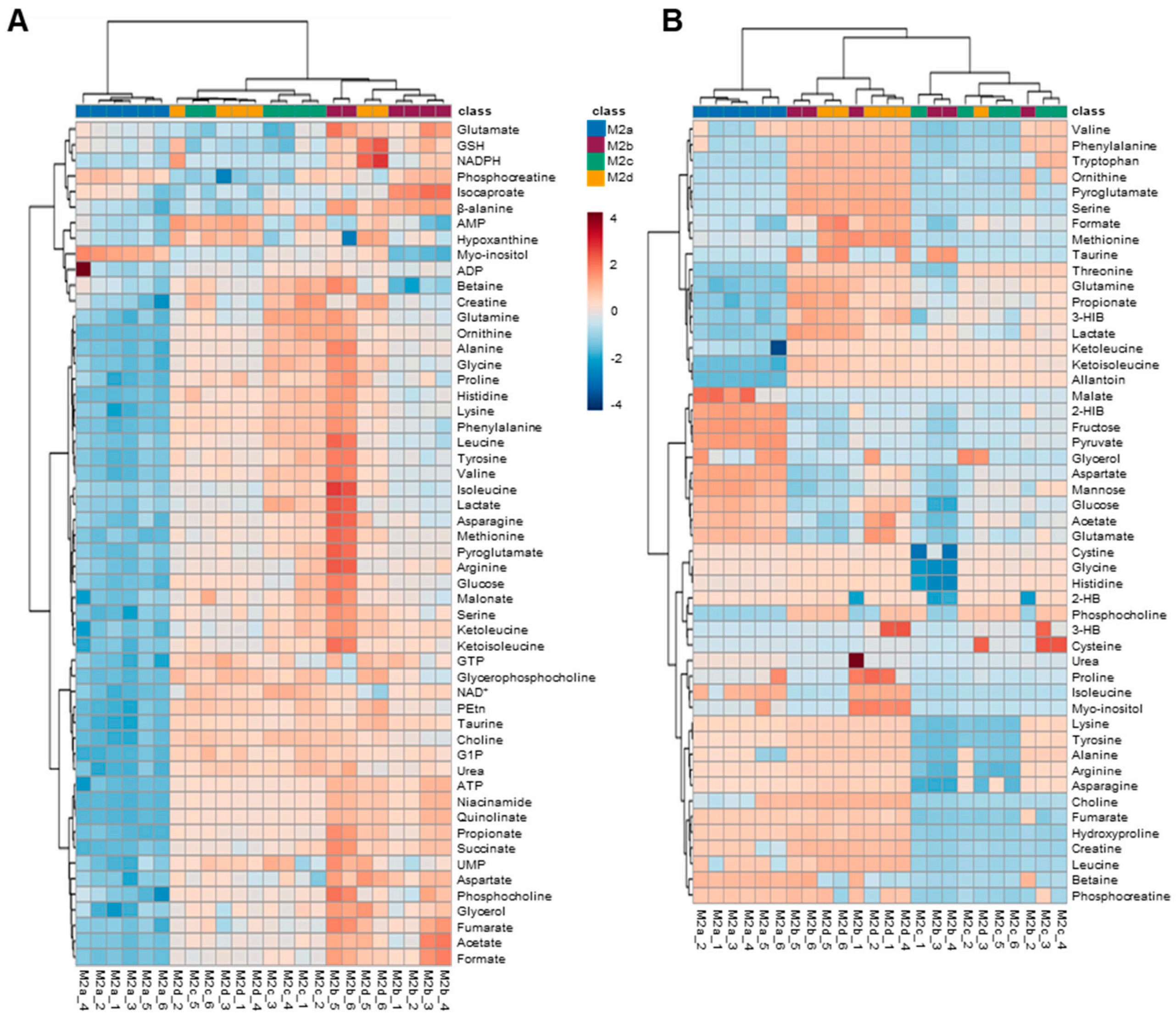

Figure S7. Hierarchical clustering analysis (HCA) and heatmap visualizations of M2a (blue), M2b (red), M2c (green), and M2d (yellow) MΦ subtype **(A)** intra- and **(B)** extracellular polar metabolite profiles. The color scales represent the scaled abundance of each metabolite, with dark red and blue indicating high and low abundance, respectively. Abbreviations denote: 2-HB, 2-hydroxybutyrate; 3-HB, 3-hydroxybutyrate; 2-HIB, 2-hydroxyisobutyrate; 3-HIB, 3-hydroxyisobutyrate; ADP, adenosine diphosphate; AMP, adenosine monophosphate; ATP, adenosine triphosphate; G1P, glucose-1-phosphate; GSH, glutathione; GTP, guanosine triphosphate; NAD+, nicotinamide adenine dinucleotide; NADPH, nicotinamide adenine dinucleotide phosphate; PEtn, phosphatidyl ethanolamine; UMP, uridine monophosphate.

Table S1. Polar metabolites identified in intracellular metabolite extracts of M2 MΦ subtypes.

Metabolite	Concentration (nmol/mg)[1]							
	M2a		M2b		M2c		M2d	
	Mean	SD	Mean	SD	Mean	SD	Mean	SD
Acetate	3.34	0.42	91.42	72.66	21.74	3.58	28.75	20.16
ADP	33.17	48.76	18.14	3.47	18.63	1.46	16.54	2.12
Alanine	2.57	0.27	11.13	6.42	10.04	1.93	7.52	0.97
AMP	7.19	1.03	7	1.51	11.9	3.25	14.5	1.6
Arginine	7.49	1.04	36.73	23.65	20.33	5.19	16.73	1.09
Asparagine	3.54	0.65	13.27	9.18	8.89	1.16	8.29	1.77
Aspartate	1.64	0.22	4.51	0.71	2.85	0.71	3.94	1.27
ATP	0.28	0.05	1.84	0.36	1.2	0.04	1.35	0.27
Betaine	2.15	0.37	2.66	1.57	3.77	0.3	2.67	0.21
Choline	0.86	0.19	5.17	2.03	8.69	0.38	6.68	0.75
Creatine	3.19	0.66	4.37	0.3	6.58	0.68	5.07	1.44
Formate	4.29	0.34	32.28	13.14	12.7	2.1	15.47	4.37
Fumarate	0.61	0.06	1.67	0.4	1.06	0.13	1.06	0.24
G1P	0.31	0.05	3.81	0.67	5.09	1.49	4.01	0.9
Glucose	4.53	1.05	104.79	114.05	52.4	30.64	44.38	6.45
Glutamate	14.83	1.82	28.7	7.07	10.75	3.1	16.14	5.22
Glutamine	9.69	1.56	24.56	10.86	34.05	7.15	20.02	4.18
Glutathione	9.62	1.22	13.73	1.7	7.96	1.73	14.43	7.35
Glycerol	0.44	0.3	7.67	5.32	3.45	0.91	5.59	6.04
Glycine	8.48	0.82	18.94	7.3	20.74	2.68	17.31	2.89
GPC	0.85	0.11	4.29	1.93	11.69	2.34	16.96	2.84
GTP	1.82	0.23	3.86	0.89	3.8	0.67	4.44	0.5
Histidine	0.63	0.05	3.79	2.93	4.26	0.85	3.25	0.31
Hypoxanthine	1.19	0.22	1.55	0.6	1.92	0.72	3.12	0.31
Isocaproate	0.39	0.19	1.8	1.14	0.24	0.07	0.23	0.1
Isoleucine	3.2	0.22	8.95	6.55	6.3	1.13	5.47	0.88
Ketoisoleucine	0.34	0.07	2.65	1.88	1.61	0.21	1.22	0.2
Ketoleucine	0.12	0.04	1.25	0.65	0.82	0.28	0.62	0.3
Lactate	143.89	16.31	448.7	317.16	387.85	101.06	240.41	12.9
Leucine	3.24	0.42	11.85	9.37	10.73	1.67	7.87	0.65
Lysine	0.79	0.22	7.2	5.73	7.55	1.19	5.49	1.01
Malonate	0.31	0.13	5.13	5.55	3.86	1.61	1.84	0.36
Methionine	0.62	0.08	2.88	1.98	1.87	0.31	1.87	0.28
myo-Inositol	51.8	7.38	19.08	7.35	26.58	1.96	26.71	7.29
NAD+	0.53	0.13	5.53	0.7	9.69	1.78	3.86	2.14
NADPH	1.08	0.08	1.84	0.36	1.2	0.04	2.7	1.7
Niacinamide	0.29	0.01	1.84	0.36	1.2	0.04	1.35	0.27
Ornithine	0.29	0.01	6	4.98	8.57	4.16	3	0.47
PEtn	15.2	2.74	61.52	6.95	95.05	13.62	106.6	19.91
Phenylalanine	0.6	0.07	4.73	4.24	5.12	0.69	3.73	0.37
Phosphocholine	7.24	1.61	16.14	5.06	12.14	0.46	11.95	0.71
Phosphocreatine	1.5	0.2	1.6	0.28	0.74	0.47	0.46	0.22
Proline	5.03	1.06	15.69	7.73	15.44	1.6	14.46	3.04
Propionate	0.61	0.06	4.38	1.95	2.43	0.17	2.9	0.72
Pyroglutamate	7.43	1.34	70.55	66.79	38.15	9.17	26.44	4.58
Quinolinate	0.29	0.01	1.84	0.36	1.2	0.04	1.35	0.27
Serine	1.99	0.53	19.13	13.11	13.49	3.65	12.61	4.17
Succinate	0.68	0.09	5.89	2.44	3.1	0.57	2.95	0.91
Taurine	36.21	5.8	144.86	5.18	139.05	12.83	154.91	27.94

Tyrosine	0.93	0.13	4.36	3.25	3.67	0.83	3.17	0.41
UMP	1.81	0.27	3.24	0.93	3.23	0.79	3.43	0.4
Urea	3.07	1.22	62.93	40.96	69.49	18.64	48.26	17.44
Valine	1.87	0.23	7.82	5.44	7.03	1.01	5.71	0.43
β-Alanine	2.14	0.46	5.87	0.33	3.04	0.88	2.91	1.32

[1]A total of 54 metabolites were identified in intracellular M2 MΦ subtype spectra and exported as μM concentrations. The data were normalized using blank NMR buffer controls, the running NMR buffer volume, and protein content to report concentrations as nmol/mg. The mean and standard deviation (SD) for each normalized metabolite is shown for the M2a, M2b, M2c and M2d MΦ subtypes. Abbreviations denote: ADP, adenosine diphosphate; AMP, adenosine monophosphate; ATP, adenosine triphosphate; G1P, glucose-1-phosphate; GPC, glycerophosphocholine; GTP, guanosine triphosphate; NAD+, nicotinamide adenine dinucleotide; NADPH, nicotinamide adenine dinucleotide phosphate; PEtn, phosphatidyl ethanolamine; UMP, uridine monophosphate.

Table S2. Polar metabolites identified in extracellular metabolite extracts of M2 MΦ subtypes.

Metabolite	Concentration (nmol/mg)[2]							
	M2a		M2b		M2c		M2d	
	Mean	SD	Mean	SD	Mean	SD	Mean	SD
2-HB	23.43	6.65	-5.34	39.43	32.73	12.94	42.50	20.80
2-HIB	-169.95	5.48	-502.18	153.69	-451.26	41.94	-519.12	47.37
3-HB	-96.50	3.52	-106.54	48.54	-21.96	21.10	-10.31	25.91
3-HIB	12.49	1.85	64.77	33.66	35.17	16.82	84.33	24.38
Acetate	-213.14	25.19	-1273.28	566.71	-693.10	285.30	-655.69	572.67
Alanine	20.73	32.54	179.31	899.81	5.30	269.47	842.94	405.05
Allantoin	-28.01	5.58	1244.65	647.21	384.92	84.82	1299.62	482.54
Arginine	788.70	181.40	2243.74	3107.49	-100.85	742.78	2363.24	1418.71
Asparagine	108.27	20.01	-98.20	993.36	-38.21	267.35	1276.55	764.56
Aspartate	-573.21	20.11	-2530.94	604.37	-1891.74	209.07	-1600.60	755.08
Betaine	7.74	2.48	-14.95	59.11	-42.67	11.75	-21.27	17.76
Choline	6.41	13.55	-75.09	177.63	-92.77	59.21	74.01	64.57
Creatine	3.80	3.33	-23.39	129.58	-85.00	23.88	66.58	59.73
Cysteine	-125.00	10.18	-231.00	65.07	-47.15	59.86	-140.46	161.95
Cystine	468.59	56.96	595.48	740.46	308.27	232.12	949.43	296.93
Formate	228.89	60.64	389.50	184.67	319.73	39.02	1049.71	310.57
Fructose	-849.80	27.33	-11208.50	4379.24	-7412.92	976.99	-8689.93	3042.82
Fumarate	9.51	1.53	3.33	7.05	-5.99	5.17	7.73	7.70
Glucose	-3.06E+04	1.46E+03	-7.45E+04	2.84E+04	-5.70E+04	2.89E+03	-4.71E+04	1.59E+04
Glutamate	-363.96	16.87	-1684.55	545.01	-1156.97	278.89	-984.70	745.81
Glutamine	731.62	116.28	4348.88	2479.66	2935.25	621.45	4928.27	1647.99
Glycerol	50.34	215.89	-1355.06	636.03	-270.21	875.76	-121.23	274.29
Glycine	501.91	105.28	834.01	1300.99	305.78	260.33	2371.19	769.92
Histidine	56.36	16.27	212.86	308.69	144.00	102.46	493.52	155.78
Hydroxyproline	75.12	24.60	-422.38	1267.25	-797.09	250.99	297.42	472.25
Hypoxanthine	0.00	0.00	-67.03	6.80	-35.97	12.42	-41.35	19.96
Isoleucine	60.25	49.94	-1495.97	1328.58	-1031.91	347.04	-153.77	454.77
Ketoisoleucine	21.34	3.28	412.75	128.09	254.50	38.20	374.04	72.40
Ketoleucine	2.81	2.67	256.94	116.22	160.66	20.49	284.83	71.19
Lactate	3.82E+04	2.38E+03	1.26E+05	2.94E+04	6.44E+04	1.27E+04	1.07E+05	3.31E+04
Leucine	19.93	32.15	-796.13	1384.01	-671.05	350.71	567.73	513.77
Lysine	209.60	18.54	263.67	577.56	-56.24	234.03	702.70	422.37
Malate	-5.69	22.51	-847.95	203.38	-468.52	112.06	-569.77	163.96
Mannose	-43.79	6.75	-687.35	162.83	-223.29	110.36	-282.76	152.36
Methionine	-15.02	8.17	-339.34	383.10	-296.41	95.88	6.21	126.49
myo-Inositol	-60.26	44.66	-1432.76	1351.03	-985.48	338.03	-209.22	497.65
Ornithine	-19.80	1.71	580.05	515.67	-185.99	159.68	534.43	365.61
Phenylalanine	-9.66	8.99	35.08	386.14	-42.66	115.28	424.21	209.71
Phosphocholine	-42.20	2.06	-39.00	79.13	28.15	10.41	55.85	36.50
Phosphocreatine	26.65	2.93	0.92	91.07	-37.60	39.10	24.76	61.38
Proline	-27.75	19.26	-909.08	908.69	-702.52	386.96	107.03	578.18
Propionate	8.27	1.83	104.82	94.64	47.78	9.90	116.00	63.44
Pyroglutamate	-386.27	147.17	-616.44	5452.74	-2276.92	1286.63	1583.49	1802.24
Pyruvate	-336.50	16.08	-1580.09	439.62	-1538.96	147.93	-1731.70	295.65
Serine	-284.36	81.68	-1001.30	3332.70	-1924.15	598.78	806.33	1076.66
Taurine	-171.82	58.65	27.40	100.51	-208.39	60.11	40.39	201.45
Threonine	-126.26	21.48	516.58	651.83	335.21	200.79	1115.18	266.43
Tryptophan	-53.91	15.29	-23.38	86.05	-4.26	40.38	57.54	45.49
Tyrosine	18.25	9.11	-2.02	484.60	-94.70	133.44	433.50	243.83

Urea	-1.15E+03	1.46E+02	-1.42E+04	1.03E+04	-1.66E+04	4.69E+03	-7.84E+03	2.65E+03
Valine	10.00	45.69	-109.29	799.76	-55.95	265.10	661.69	340.03

[2]A total of 51 metabolites were identified in extracellular M2 MΦ subtype spectra and exported as μM concentrations. The data were normalized using sham media controls, the running NMR buffer volume, and protein content to report concentrations as nmol/mg. The mean and standard deviation (SD) for each normalized metabolite is shown for the M2a, M2b, M2c and M2d MΦ subtypes. Abbreviations denote: 2-HB, 2-hydroxybutyrate; 2-HIB, 2-hydroxyisobutyrate; 3-HB, 3-hydroxybutyrate; 3-HIB, 3-hydroxyisobutyrate.

Table S3. One-way parametric ANOVA analysis of intracellular M2a, M2b, M2c, and M2d MΦ metabolite levels with Tukey's post-hoc analysis.

Name	f.value	p.value	-log10(p)	FDR	Tukey's Post-Hoc Tests
G1P	175.78	1.54E-14	13.81	6.07E-13	M2b-M2a; M2c-M2a; M2d-M2a
PEtn	165.70	2.72E-14	13.57	6.07E-13	M2b-M2a; M2c-M2a; M2d-M2a; M2c-M2b; M2d-M2b
Niacinamide	157.25	4.50E-14	13.35	6.07E-13	M2b-M2a; M2c-M2a; M2d-M2a; M2c-M2b; M2d-M2b
Quinolinate	157.25	4.50E-14	13.35	6.07E-13	M2b-M2a; M2c-M2a; M2d-M2a; M2c-M2b; M2d-M2b
Taurine	151.21	6.54E-14	13.19	7.06E-13	M2b-M2a; M2c-M2a; M2d-M2a
GPC	140.96	1.28E-13	12.89	1.15E-12	M2b-M2a; M2c-M2a; M2d-M2a; M2c-M2b; M2d-M2b
ATP	115.90	8.18E-13	12.09	6.31E-12	M2b-M2a; M2c-M2a; M2d-M2a; M2c-M2b
Choline	112.88	1.05E-12	11.98	7.08E-12	M2b-M2a; M2c-M2a; M2d-M2a; M2c-M2b
Urea	64.22	1.92E-10	9.72	1.15E-09	M2b-M2a; M2c-M2a; M2d-M2a
Succinate	52.62	1.13E-09	8.95	6.12E-09	M2b-M2a; M2c-M2a; M2d-M2a; M2c-M2b; M2d-M2b
NAD+	51.84	1.29E-09	8.89	6.35E-09	M2b-M2a; M2c-M2a; M2d-M2a; M2d-M2c
Propionate	48.52	2.31E-09	8.64	1.04E-08	M2b-M2a; M2c-M2a; M2d-M2a; M2c-M2b
Formate	41.42	9.07E-09	8.04	3.77E-08	M2b-M2a; M2c-M2a; M2d-M2a; M2c-M2b; M2d-M2b
Ornithine	40.99	9.92E-09	8.00	3.83E-08	M2b-M2a; M2c-M2a; M2d-M2a; M2d-M2c
Lysine	30.82	1.07E-07	6.97	3.86E-07	M2b-M2a; M2c-M2a; M2d-M2a
Ketoleucine	28.20	2.19E-07	6.66	7.41E-07	M2b-M2a; M2c-M2a; M2d-M2a
GTP	26.46	3.64E-07	6.44	1.16E-06	M2b-M2a; M2c-M2a; M2d-M2a
Serine	25.66	4.64E-07	6.33	1.39E-06	M2b-M2a; M2c-M2a; M2d-M2a
Ketoisoleucine	24.99	5.71E-07	6.24	1.62E-06	M2b-M2a; M2c-M2a; M2d-M2a
Histidine	24.02	7.76E-07	6.11	2.10E-06	M2b-M2a; M2c-M2a; M2d-M2a
Fumarate	23.08	1.06E-06	5.98	2.72E-06	M2b-M2a; M2c-M2a; M2d-M2a; M2c-M2b; M2d-M2b
Alanine	22.74	1.18E-06	5.93	2.91E-06	M2b-M2a; M2c-M2a; M2d-M2a
Phenylalanine	21.98	1.53E-06	5.81	3.60E-06	M2b-M2a; M2c-M2a; M2d-M2a
Glutamine	19.53	3.70E-06	5.43	8.33E-06	M2b-M2a; M2c-M2a; M2d-M2a; M2d-M2c
Acetate	18.84	4.81E-06	5.32	1.04E-05	M2b-M2a; M2c-M2a; M2d-M2a
Aspartate	18.01	6.67E-06	5.18	1.39E-05	M2b-M2a; M2c-M2a; M2d-M2a; M2c-M2b
Proline	17.71	7.52E-06	5.12	1.42E-05	M2b-M2a; M2c-M2a; M2d-M2a
Glycine	17.68	7.61E-06	5.12	1.42E-05	M2b-M2a; M2c-M2a; M2d-M2a
myo-Inositol	17.66	7.65E-06	5.12	1.42E-05	M2b-M2a; M2c-M2a; M2d-M2a
Valine	16.79	1.10E-05	4.96	1.97E-05	M2b-M2a; M2c-M2a; M2d-M2a
Malonate	16.65	1.16E-05	4.93	2.03E-05	M2b-M2a; M2c-M2a; M2d-M2a
Glucose	16.40	1.29E-05	4.89	2.18E-05	M2b-M2a; M2c-M2a; M2d-M2a
Arginine	15.73	1.73E-05	4.76	2.83E-05	M2b-M2a; M2c-M2a; M2d-M2a; M2d-M2b
Methionine	15.20	2.18E-05	4.66	3.47E-05	M2b-M2a; M2c-M2a; M2d-M2a
AMP	15.04	2.35E-05	4.63	3.62E-05	M2c-M2a; M2d-M2a; M2c-M2b; M2d-M2b
Tyrosine	14.44	3.08E-05	4.51	4.55E-05	M2b-M2a; M2c-M2a; M2d-M2a
Glycerol	14.42	3.12E-05	4.51	4.55E-05	M2b-M2a; M2c-M2a; M2d-M2a
Pyroglutamate	14.12	3.59E-05	4.44	5.11E-05	M2b-M2a; M2c-M2a; M2d-M2a
Creatine	14.00	3.79E-05	4.42	5.25E-05	M2b-M2a; M2c-M2a; M2d-M2a; M2c-M2b
Glutamate	13.41	5.05E-05	4.30	6.81E-05	M2b-M2a; M2c-M2b; M2d-M2b
Beta-Alanine	13.11	5.83E-05	4.23	7.68E-05	M2b-M2a; M2c-M2b; M2d-M2b
Phosphocholine	12.54	7.75E-05	4.11	9.97E-05	M2b-M2a; M2c-M2a; M2d-M2a
Phosphocreatine	12.30	8.78E-05	4.06	1.10E-04	M2c-M2a; M2d-M2a; M2c-M2b; M2d-M2b

UMP	9.98	3.13E-04	3.50	3.84E-04	M2b-M2a; M2c-M2a; M2d-M2a
Asparagine	9.53	4.09E-04	3.39	4.91E-04	M2b-M2a; M2c-M2a; M2d-M2a
Leucine	9.41	4.39E-04	3.36	5.15E-04	M2b-M2a; M2c-M2a; M2d-M2a
Lactate	7.67	1.33E-03	2.88	1.53E-03	M2b-M2a; M2c-M2a
Isocaproate	6.92	2.22E-03	2.65	2.50E-03	M2b-M2a; M2c-M2b; M2d-M2b
Hypoxanthine	6.84	2.36E-03	2.63	2.60E-03	M2d-M2a; M2d-M2b
NADPH	4.64	1.27E-02	1.89	1.38E-02	M2d-M2a
Isoleucine	4.56	1.36E-02	1.87	1.44E-02	M2b-M2a; M2c-M2a
Glutathione	3.91	2.39E-02	1.62	2.48E-02	M2c-M2b
Betaine	3.75	2.74E-02	1.56	2.80E-02	M2c-M2a

A total of 53 metabolites were identified through the ANOVA analysis of the intracellular M2 MΦ subtypes to have metabolite level differences that discriminated M2a, M2b, M2c, and M2d subtypes from each other. Abbreviations denote: AMP, adenosine monophosphate; ATP, adenosine, triphosphate; G1P, glucose-1-phosphate; GPC, glycerophosphocholine; GTP, guanosine triphosphate; NAD+, nicotinamide adenine dinucleotide; NADPH, nicotinamide adenine dinucleotide phosphate; PEtn, phosphatidyl ethanolamine; UMP, uridine monophosphate.

Table S4. One-way parametric ANOVA analysis of extracellular M2a, M2b, M2c, and M2d MΦ metabolite levels with Tukey's post-hoc analysis.

Name	f.value	p.value	-log10(p)	FDR	Tukey's Post-Hoc Tests
Allantoin	3379.80	3.23E-27	26.49	1.62E-25	M2b-M2a; M2c-M2a; M2d-M2a; M2c-M2b; M2d-M2c
Ketoisoleucine	216.02	2.11E-15	14.68	5.28E-14	M2b-M2a; M2c-M2a; M2d-M2a; M2c-M2b; M2d-M2c
Fructose	104.37	2.19E-12	11.66	3.19E-11	M2b-M2a; M2c-M2a; M2d-M2a
Pyruvate	102.68	2.55E-12	11.59	3.19E-11	M2b-M2a; M2c-M2a; M2d-M2a
Mannose	44.35	5.05E-09	8.30	5.05E-08	M2b-M2a; M2c-M2a; M2d-M2a; M2c-M2b; M2d-M2b
2-HIB	37.51	2.11E-08	7.68	1.75E-07	M2b-M2a; M2c-M2a; M2d-M2a
Aspartate	27.68	2.55E-07	6.59	1.82E-06	M2b-M2a; M2c-M2a; M2d-M2a; M2d-M2b
Lactate	26.47	3.63E-07	6.44	2.27E-06	M2b-M2a; M2c-M2a; M2d-M2a; M2c-M2b; M2d-M2c
Glutamine	17.94	6.85E-06	5.16	3.81E-05	M2b-M2a; M2c-M2a; M2d-M2a
3-HIB	16.86	1.06E-05	4.97	5.32E-05	M2b-M2a; M2c-M2a; M2d-M2a; M2d-M2c
Propionate	16.49	1.24E-05	4.91	5.65E-05	M2b-M2a; M2c-M2a; M2d-M2a
Betaine	16.28	1.36E-05	4.87	5.67E-05	M2c-M2a; M2d-M2a; M2c-M2b; M2d-M2b
Malate	15.85	1.64E-05	4.79	6.30E-05	M2b-M2a; M2c-M2a; M2d-M2a
Formate	15.26	2.13E-05	4.67	7.60E-05	M2d-M2a; M2d-M2b; M2d-M2c
Phosphocholine	10.87	1.88E-04	3.73	6.28E-04	M2c-M2a; M2d-M2a; M2c-M2b
Ketoleucine	10.60	2.19E-04	3.66	6.85E-04	M2b-M2a; M2c-M2a; M2d-M2a
Threonine	10.40	2.46E-04	3.61	7.22E-04	M2b-M2a; M2c-M2a; M2d-M2a
Fumarate	10.04	3.02E-04	3.52	8.40E-04	M2c-M2a; M2c-M2b; M2d-M2c
2-HB	9.09	5.32E-04	3.27	1.33E-03	M2b-M2a; M2c-M2b; M2d-M2b
Glucose	9.06	5.44E-04	3.26	1.33E-03	M2b-M2a; M2c-M2a
Methionine	9.02	5.57E-04	3.25	1.33E-03	M2d-M2a; M2d-M2b; M2d-M2c
Pyroglutamate	8.85	6.20E-04	3.21	1.41E-03	M2b-M2a; M2d-M2a; M2c-M2b; M2d-M2c
Hydroxyproline	8.67	6.94E-04	3.16	1.51E-03	M2c-M2a; M2d-M2c
Glutamate	8.28	8.88E-04	3.05	1.85E-03	M2b-M2a; M2c-M2a
Acetate	7.54	1.45E-03	2.84	2.86E-03	M2b-M2a; M2c-M2a; M2d-M2b
Serine	7.50	1.49E-03	2.83	2.86E-03	M2d-M2a; M2d-M2c
Ornithine	6.29	3.52E-03	2.45	6.52E-03	M2b-M2a; M2d-M2a; M2d-M2c
Isoleucine	5.84	4.90E-03	2.31	8.75E-03	M2b-M2a; M2c-M2a
Creatine	5.58	6.00E-03	2.22	1.04E-02	M2c-M2a; M2d-M2c
Leucine	5.41	6.87E-03	2.16	1.14E-02	M2c-M2a; M2d-M2c
Choline	4.53	1.40E-02	1.85	2.25E-02	M2d-M2c
Tryptophan	4.38	1.58E-02	1.80	2.48E-02	M2d-M2a
Taurine	4.08	2.06E-02	1.69	3.12E-02	

A total of 33 metabolites were identified through the ANOVA analysis of the extracellular M2 MΦ subtypes to have metabolite level differences that discriminated M2a, M2b, M2c, and M2d subtypes from each other. Abbreviations denote: 2-HB, 2-hydroxybutyrate; 2-HIB, 2-hydroxyisobutyrate; 3-HIB, 3-hydroxyisobutyrate.

Table S5. One-way parametric ANOVA analysis of intracellular M2b, M2c, and M2d MΦ metabolite levels with Tukey's post-hoc analysis.

Name	f.value	p.value	-log10(p)	FDR	Tukey's Post-Hoc Tests
GPC	36.19	1.82E-06	5.74	9.83E-05	M2c-M2b; M2d-M2b
PEtn	18.89	7.98E-05	4.10	2.16E-03	M2c-M2b; M2d-M2b
Glutamate	15.75	2.06E-04	3.69	3.71E-03	M2c-M2b; M2d-M2b
AMP	13.90	3.85E-04	3.41	5.20E-03	M2c-M2b; M2d-M2b
β-Alanine	11.29	1.02E-03	2.99	1.10E-02	M2c-M2b; M2d-M2b
Isocaproate	10.34	1.50E-03	2.82	1.14E-02	M2c-M2b; M2d-M2b
Phosphocreatine	10.22	1.58E-03	2.80	1.14E-02	M2c-M2b; M2d-M2b
Formate	10.06	1.69E-03	2.77	1.14E-02	M2c-M2b; M2d-M2b
Choline	8.50	3.41E-03	2.47	1.58E-02	M2c-M2b
NAD+	8.35	3.65E-03	2.44	1.58E-02	M2d-M2c
ATP	8.26	3.81E-03	2.42	1.58E-02	M2c-M2b; M2d-M2b
Niacinamide	8.26	3.81E-03	2.42	1.58E-02	M2c-M2b; M2d-M2b
Quinolinate	8.26	3.81E-03	2.42	1.58E-02	M2c-M2b; M2d-M2b
Creatine	7.61	5.22E-03	2.28	2.02E-02	M2c-M2b; M2d-M2c
Fumarate	7.36	5.94E-03	2.23	2.14E-02	M2c-M2b; M2d-M2b
Succinate	6.74	8.14E-03	2.09	2.75E-02	M2c-M2b; M2d-M2b

A total of 16 metabolites were identified through the ANOVA analysis of the intracellular M2 MΦ subtypes to have metabolite level differences that discriminated M2b, M2c, and M2d subtypes from each other. Abbreviations denote: AMP, adenosine monophosphate; ATP, adenosine triphosphate; GPC, glycerophosphocholine; NAD+, nicotinamide adenine dinucleotide; PEtn, phosphatidyl ethanolamine.

Table S6. One-way parametric ANOVA analysis of extracellular M2b, M2c, and M2d MΦ metabolite levels with Tukey's post-hoc analysis.

Name	f.value	p.value	-log10(p)	FDR	Tukey's Post-Hoc Tests
Formate	13.29	4.77E-04	3.32	1.75E-02	M2d-M2b; M2d-M2c
Allantoin	11.44	9.59E-04	3.02	1.75E-02	M2c-M2b; M2d-M2c
Mannose	11.27	1.03E-03	2.99	1.75E-02	M2c-M2b; M2d-M2b
Methionine	9.50	2.16E-03	2.67	2.52E-02	M2d-M2b; M2d-M2c
2-HB	9.20	2.47E-03	2.61	2.52E-02	M2c-M2b; M2d-M2b
Lactate	8.59	3.26E-03	2.49	2.77E-02	M2c-M2b; M2d-M2c
Hypoxanthine	7.40	5.81E-03	2.24	2.88E-02	M2c-M2b; M2d-M2b
Leucine	7.35	5.96E-03	2.23	2.88E-02	M2d-M2c
Betaine	7.29	6.13E-03	2.21	2.88E-02	M2c-M2b; M2d-M2b
Pyroglutamate	7.28	6.16E-03	2.21	2.88E-02	M2c-M2b; M2d-M2c
Fumarate	7.26	6.24E-03	2.20	2.88E-02	M2c-M2b; M2d-M2c
Creatine	6.82	7.83E-03	2.11	2.88E-02	M2d-M2c
Malate	6.67	8.48E-03	2.07	2.88E-02	M2c-M2b
Hydroxyproline	6.56	8.96E-03	2.05	2.88E-02	M2d-M2c
Choline	6.56	8.96E-03	2.05	2.88E-02	M2d-M2c
Serine	6.55	9.02E-03	2.04	2.88E-02	M2d-M2c
Ketoisoleucine	13.29	4.77E-04	3.32	1.75E-02	M2c-M2b; M2d-M2c

A total of 17 metabolites were identified through the ANOVA analysis of the extracellular M2 MΦ subtypes to have metabolite level differences that discriminated M2b, M2c, and M2d subtypes from each other. Abbreviations denote: 2-HB, 2-hydroxybutyrate.
